# EFhd2 regulates microtubule-dependent mitochondrial fitness at the B cell immune synapse

**DOI:** 10.26508/lsa.202603876

**Published:** 2026-11-04

**Authors:** Leonie Weckwerth, Benjamin Schmid, Philipp Tripal, Ann-Kathrin Himmelreich, Jana Thomas, Katharina Pracht, Sophia Urbanczyk, Hannah Allnach, Anna Gottwald, Georg Schett, Ralph Palmisano, Dirk Mielenz

**Affiliations:** 1 Department of Translational Immunology, Department of Medicine 3 - Rheumatology and Immunology, Nikolaus-Fiebiger Center for Molecular Medicine, Friedrich-Alexander-Universität (FAU) Erlangen-Nürnberg and Uniklinikum Erlangen , Erlangen, Germany; 2 Deutsches Zentrum Immuntherapie, Friedrich-Alexander-Universität (FAU) Erlangen-Nürnberg and Uniklinikum Erlangen , Erlangen, Germany; 3 Optical Imaging Competence Centre Erlangen (FAU OICE), Friedrich-Alexander-Universität (FAU) Erlangen-Nürnberg , Erlangen, Germany; 4 FAU Profile Center Immunomedicine, FAU Erlangen-Nürnberg , Erlangen, Germany; 5 Department of Medicine 3 - Rheumatology and Immunology, Friedrich-Alexander-Universität (FAU) Erlangen-Nürnberg and Uniklinikum Erlangen , Erlangen, Germany

## Abstract

Immobilized antigen induces an immune synapse, which is supported by Swiprosin-1/EFhd2 (EFhd2) in B and T cells. Here, we find that EFhd2 regulates mitochondrial fitness in the B cell synapse. Whereas activated EFhd2-deficient (KO) B cells show higher mitochondrial mass than wildtype (WT) B cells, this potential advantage reverses on coated anti-IgM antibodies: activated WT B cells enhance basal and maximal respiration as well as proton leak, with large increments of mitochondrial spare capacity, but EFhd2KO B cells fail to do so. Actin destabilization, but much more so microtubule destabilization, impairs mitochondrial up-regulation in WT, but not in EFhd2KO B cells. Conversely, microtubule stabilization, and to a lesser extent actin stabilization, restore mitochondrial spare capacity in EFhd2KO B cells at the BCR synapse. EFhd2KO B cells fail to coordinate the microtubule organization center, mitochondria and IgM BCRs upon synapse formation. Hence, microtubule-dependent mitochondrial organization at the immune synapse coordinates mitochondrial fitness in B cells via EFhd2.

## Abstract (precis)

Formation of B-cell synapses on immobilized anti-IgM antibodies in B-cell receptor (BCR)- and CD40/interleukin-4 (IL-4)-activated B cells increases mitochondrial spare capacity. This process relies on microtubule stabilization and mitochondrial reorganization, both of which are mediated by EFhd2.

## Introduction

B cells can recognize a multitude of diverse soluble or membrane-bound antigens owing to the unique structural organization of the B cell receptor (BCR) (Gold & Reth, 2019). Thus, initial BCR activation can be achieved by soluble antigen or by immobilized antigen on antigen-presenting cells (APCs), such as dendritic cells (DC) in the B cell follicle (Wykes et al, 1998; Batista et al, 2001). This variability ensures efficient discrimination and elimination of different kinds of pathogenic structures and has functional consequences: For instance, repetitive immobile antigen arrays induce stronger anti-viral humoral immune responses than soluble antigens (Amanna et al, 2007; Perotti & Perez, 2019). Activated antigen-specific B cells remain motile within lymphoid follicles and can undergo repeated BCR engagement whereas sampling antigen displayed on follicular antigen-presenting surfaces (Harwood & Batista, 2010; Crotty, 2011; Victora & Nussenzweig, 2022). Although repeated BCR signaling enhances antigen uptake and promotes activation, BCR engagement alone is generally insufficient to drive full activation and differentiation of B cells responding to T-dependent protein antigens. Sustained responses require cognate T-cell help via CD40L and IL-4 which lead different routes of plasma cell differentiation, via extrafollicular responses or germinal centers (GC), depending on initial BCR affinity (Young & Brink, 2021). Sufficiently co-stimulated B cells can seed GC around the follicular DC (FDC) network (Allen et al, 2004; Mintz & Cyster, 2020; Victora & Nussenzweig, 2022). In GC, antibody (Ab) affinity maturation occurs via clonal expansion of B cells, followed by iterative cycles of somatic hypermutation (SHM) of Ab variable gene segments and positive selection (Steiner & Eisen, 1967; Eisen, 2014; Victora & Nussenzweig, 2022). To this end, FDC represent antigen in the form of Ag-Ab immune complexes or complement-tagged antigens generated earlier. The light zone (LZ) GC B cells recognize immobilized antigen on FDC via their BCR and specialized synapses (Natkanski et al, 2013; Nowosad et al, 2016). These synapses form despite the low BCR surface expression in GC B cells (Paus et al, 2006; Chan et al, 2009; Kwak et al, 2018) and assess the BCRs for preserved and, at best, improved antigen binding (Hauser et al, 2007; Beltman et al, 2011; Victora & Nussenzweig, 2022). This process involves coupling of the BCR to the Actin-Myosin system and force generation which is proportional to BCR affinity, favoring high affine B cells to acquire and present antigen to follicular T helper cells (TFH) (Natkanski et al, 2013; Victora & Nussenzweig, 2022). Thereby, the LZ B cells undergo positive selection signals via BCR signaling (Inoue et al, 2024), metabolic remodeling (Inoue et al, 2024) and co-stimulation, unleashing anabolic pathways (Ersching et al, 2017). These signals also promote recycling of LZ B cells to the dark zone (DZ) or, finally, plasma cell and memory B cell (MBC) differentiation (Ersching et al, 2017; Toboso-Navasa et al, 2020). Notably, catabolic metabolism can be an important upstream prerequisite of mechanistic target of rapamycin (mTOR) activity, which drives anabolic processes in activated B cells (Urbanczyk et al, 2022; Bierling et al, 2024). In alignment, regulation of genes required for oxidative phosphorylation (OxPhos) in LZ GC B cells, as well as transcription factor A (TFAM) activity and mitochondrial fatty acid oxidation support GC responses and antibody affinity maturation dynamically (Weisel et al, 2020; Chen et al, 2021; Urbanczyk et al, 2022; Yazicioglu et al, 2023). Interestingly, mitochondrial mass and their spatial organization are more pronounced in LZ than in DZ GC B cells, suggesting that the location of GC B cells or their contact with FDC control respiratory chain activity (Yazicioglu et al, 2023), in line with BCR affinity-coupled OxPhos gene expression (Chen et al, 2021). These data infer that the organization of the LZ B-FDC synapse via the BCR directs mitochondrial function. Because the Actin-Myosin system is involved in the LZ B-FDC synapse, this modulation of mitochondrial function could occur via the cytoskeleton. Importantly, B cell synapses are not only formed in GC B cells but also in B cells in the follicle that contact DC (Wykes et al, 1998; Batista et al, 2001). They therefore present general structures guiding anabolic B cell expansion. Yet, how synapse formation is linked to metabolic reprogramming in B cells is currently not clear (Carrasco, 2023). Previously we have shown that the speed and velocity of GC B cells are proportional to GC plasma cell output (Reimer et al, 2020). To show this, we have used B cells from mice deficient for the Actin-binding and bundling protein Swiprosin-1/EFhd2 (EFhd2). EFhd2ʼs Actin-bundling activity is enhanced by binding of Ca^2+^ to its EF-hands (Huh et al, 2013; Kwon et al, 2013; Park et al, 2016). Besides binding F-Actin, EFhd2 also interacts with the microtubule (MT) system by slowing down kinesin-mediated anterograde transport on microtubules (Purohit et al, 2014; Mielenz & Gunn-Moore, 2016). Consistent with its role in coordinating cytoskeletal dynamics, loss of EFhd2 increases Actin dynamics at the expense of B cell polarization. EFhd2KO B cells form smaller and less organized synapses with FDC in vivo, as shown by 2-Photon microscopy, and exhibit competitive disadvantages in the GC (Reimer et al, 2020). Together, these data suggest that EFhd2 supports the (metabolic) fitness of GC B cells via the immune synapse. Here, we tested whether B cells change their metabolic properties upon BCR mediated synapse formation and whether EFhd2 modulates this process. We reveal that the BCR can sense antigenic nature via EFhd2-mediated microtubule stabilization, and translate this signal into mitochondrial fitness.

## Results

### Increased mitochondrial mass in EFhd2KO B cells activated in suspension

**Figure 1.**
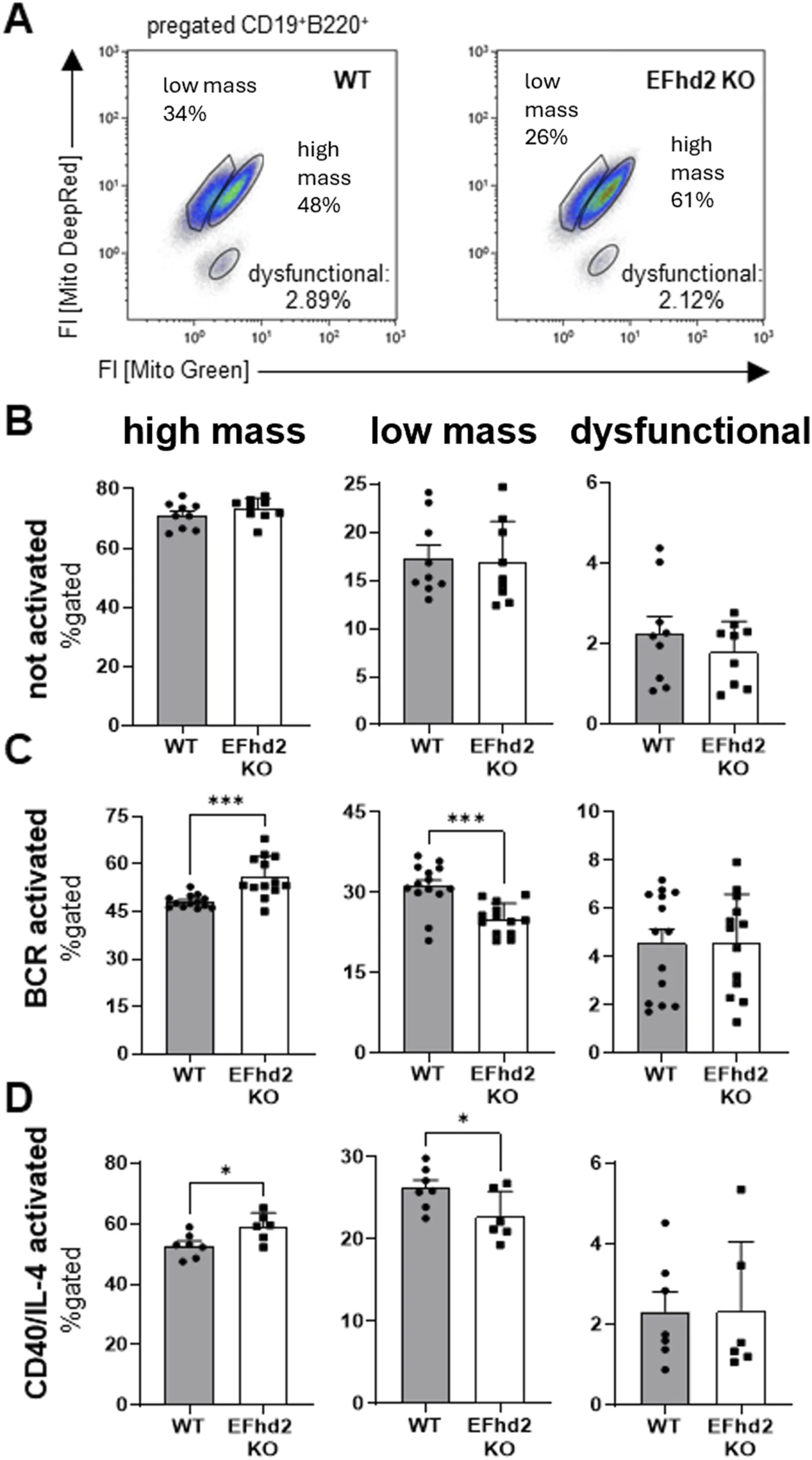
Effect of EFhd2 loss on mitochondria in B cells. **(A)** 24 h BCR-activated splenic B cells from WT and EFhd2 KO mice were stained with using Mitotracker Green (Mito Green) and Mitotracker DeepRed (Mito DeepRed). Representative gating strategy to depict frequencies of cells with high mitochondrial mass (Mitotracker Deep Red^high^, Mitotracker Green^high^), low mitochondrial mass (Mitotracker Deep Red^high^, Mitotracker Green^low^) and dysfunctional mitochondria (Mitotracker Deep Red^low^, Mitotracker Green^pos^). **(B)** Non-activated B cells, C, 24 h anti-IgM or D, 24 h anti-CD40 + IL-4 activated B cells. Data are presented as mean ± SEM; each dot represents one mouse; statistics: B. N = 3, n = 9. **(C)** N = 6, n = 13-14. **(D)** N = 2, n = 6–7; significance was analyzed using unpaired *t* test or Mann-Whitney test (*P-*values* <*0.5. *P < 0.05, ***P < 0.002). N: number of experiments, n: individual mice.

**Figure S1.**
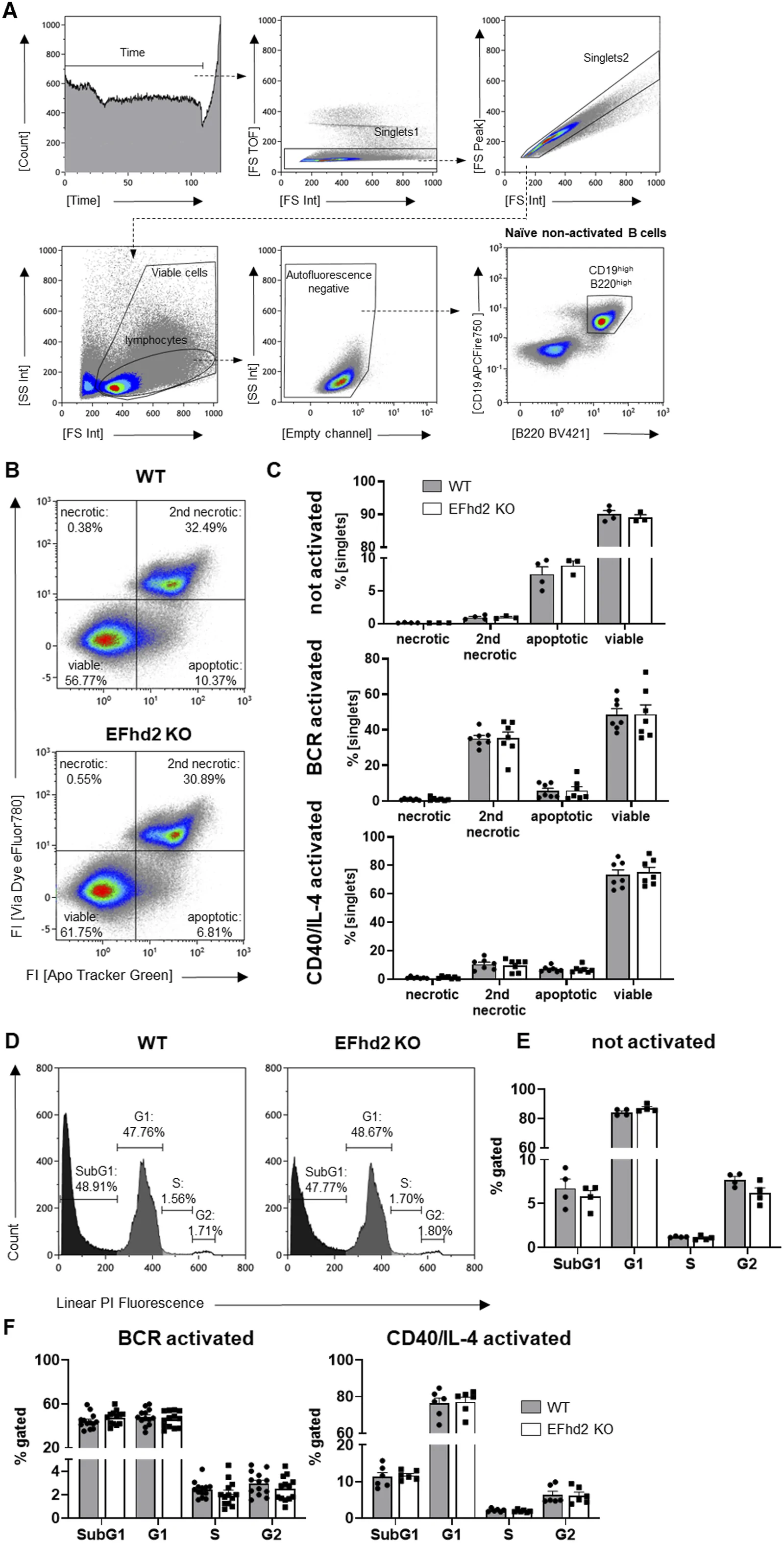
Flow cytometry and fitness of activated WT and EFhd2KO B cells. **(A)** Representative flow cytometry gating strategy. **(B)** Viability and apoptosis of activated WT and EFhd2 KO B cells assessed by staining with eFluor780 and ApoTracker Green. **(C)** Quantification of eFluor780 and ApoTracker Green staining of non-activated (d0) and 24 h anti-IgM or anti-CD40/IL-4 stimulated WT and EFhd2 KO B cells, N = 1 (not activated) and N = 2 (activated), n = 3–7. **(D)** Cell cycle progression was analyzed by staining WT and EFhd2 KO B cells with Triton X-100/Citrate/PI and nuclei were analyzed by flow cytometry. Representative histograms show gating used to distinguish subG1, G1, S, and G2 phases. **(E, F)** Percentages of WT and EFhd2 KO B cells in each phase of the cell cycle. Data are presented as mean −/+ SEM; each dot represents one mouse. N = 2–4, n = 4–13. Two-way ANOVA. No significant differences detected. N, number of experiments; n, individual mice.

We addressed mitochondrial abundance and membrane potential (ψ_Mito_) in splenic B cells of WT and EFhd2KO mice following soluble stimulation by flow cytometry (Fig 1; complete gating strategy in Fig S1A). Cells were classified by mitochondrial mass (high or low; Mitotracker Deep Red^high^, Mitotracker Green^high^ or ^low^, respectively) or as cells containing depolarized mitochondria (Mitotracker Deep Red^low^, Mitotracker Green^pos^) (Jang et al, 2015; Fig 1A). There were no signs of mitochondrial alterations in resting EFhd2KO cells (Fig 1B). However, the frequency of cells with high mitochondrial mass was higher in EFhd2KO B cells activated by soluble anti-IgM or anti-CD40 antibodies plus IL-4 (CD40/IL-4) (Fig 1C and D). The proportion of cells with depolarized mitochondria was unchanged. Thus, loss of EFhd2 leads to more activated cells with high mitochondrial mass.

### EFhd2 elevates respiration and mitochondrial spare capacity in plate-bound anti-IgM stimulated B cells

**Figure 2.**
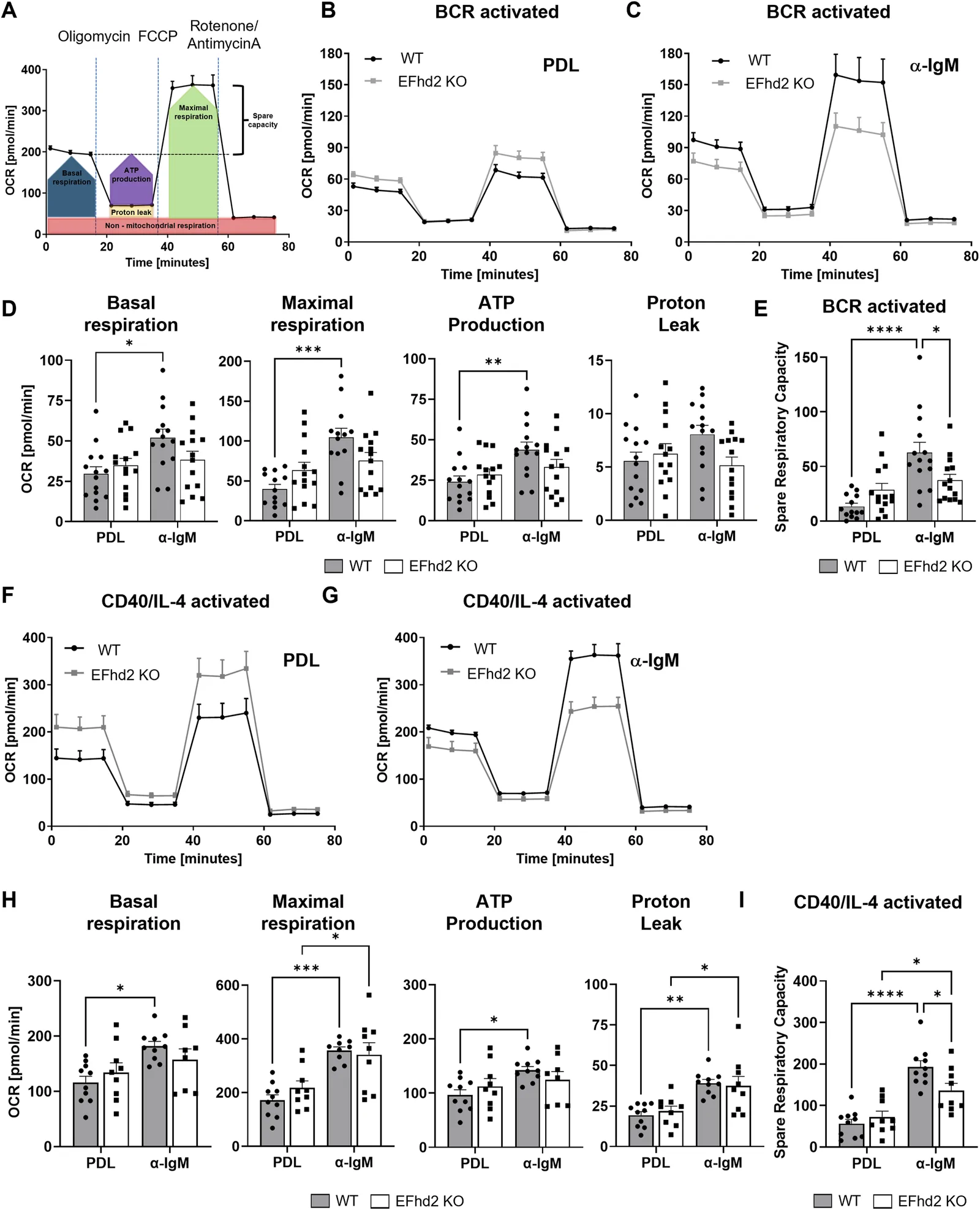
EFhd2 increases respiration, proton leak and spare capacity upon plate-bound IgM activation. **(A)** Schematic of the experimental setup. Oxygen consumption rate (OCR) was measured before and after injection of Oligomycin, FCCP and Rotenone + Antimycin A using the Seahorse Wave Mito Stress Test (MST) protocol. **(B, C)** Representative extracellular flux analysis of 24 h anti-IgM Ab activated splenic B cells attached to poly-D-lysine (PDL) or anti-IgM antibodies. Symbols represent means of two mice, each of three to four replicate wells, mean ± SEM. **(D)** Calculated OCR of basal respiration, maximal respiration, ATP production and proton leak of 24 h anti-IgM activated B cells attached to PDL or anti-IgM antibodies. **(E)** Calculated spare respiratory capacity (SPRC). **(D, E)** Data are presented as mean ± SEM; each dot represents one mouse; statistics: N = 7, n = 14; Two-way ANOVA (*P*-values <0.5. *P < 0.05, **P < 0.01, ***P < 0.002, ****P < 0.0004). **(F)** Representative extracellular flux analysis of 24 h anti-CD40/IL-4 activated splenic B cells attached to PDL or (G). anti-IgM antibodies. Symbols represent means of two mice, each of three to four replicate wells, mean ± SEM. **(H)** Calculated OCR of basal respiration, maximal respiration, ATP production and proton leak of 24 h anti-CD40/IL-4 activated B cells attached to PDL or anti-IgM Ab. Data are presented as mean ± SEM; each dot represents one mouse; N = 3, n = 9–10; Two-way ANOVA (*P*-values <0.5. *P < 0.05, **P < 0.01, ***P < 0.002, ****P < 0.0004). **(I)** Calculated SPRC. Data are presented as mean ± SEM; each dot represents one mouse; N = 3, n = 9–10; Two-way ANOVA (*P*-values <0.5. *P < 0.05, **P < 0.01, ***P < 0.002, ****P < 0.0004). N: number of experiments, n: individual mice.

**Figure S2.**
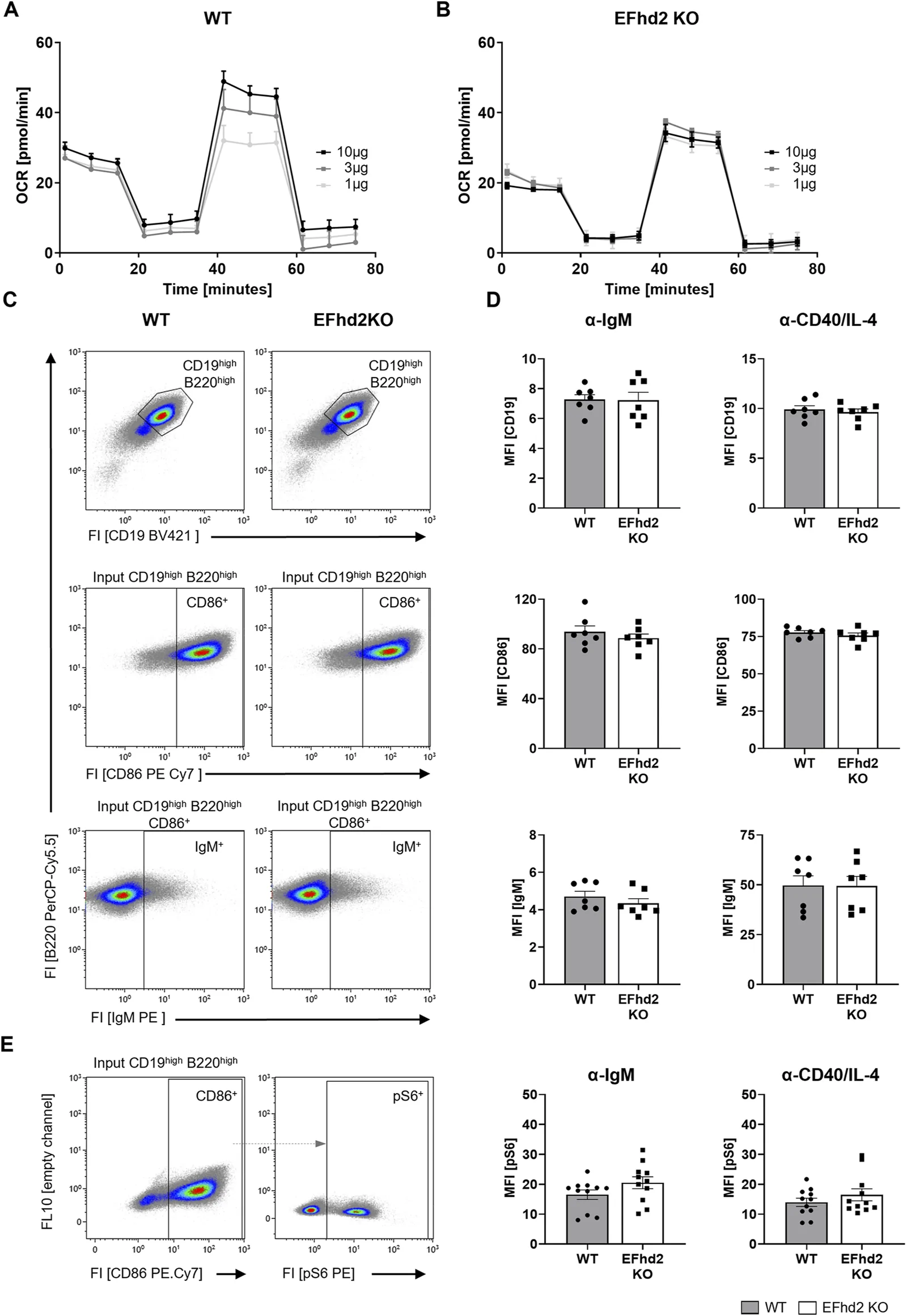
Titration of anti-BCR antibodies on Seahorse plates and similar activation of WT and EFhd2KO B cells. **(A, B)** Extracellular flux analysis of 24 h anti-IgM Ab activated splenic WT (A) and EFhd2 KO (B) B cells attached to anti-IgM Ab with different coating concentrations. The basal oxygen consumption rate (OCR) was measured before and after injection of Oligomycin, FCCP and Rotenone + Antimycin A using the Seahorse Wave Mito Stress Test (MST) protocol. Symbols represent means of two mice, each of three to four replicate wells, mean ± SEM. **(C)** Splenic B cells from wild type (WT) and EFhd2 KO mice were stimulated with anti-IgM or anti-CD40 Ab plus IL-4 for 24 h and stained with antibodies directed against CD19, B220, IgM, and CD86. Representative dot plots for anti-IgM stimulated cells. **(D)** Surface abundance of CD19, CD86 and IgM, quantified as mean fluorescence intensity (MFI). **(E)** Intracellular phosphorylated S6 (pS6) staining in activated (CD19^high^ B220^high^ CD86^+^) WT and EFhd2 KO B cells, quantified by MFI using flow cytometry. Data are presented as mean ± SEM; each dot represents one mouse; statistics: (D). N = 2, n = 7; (C). N = 4, n = 11, unpaired *t* test. If not indicated otherwise, the differences were not significant (n.s.). N, number of experiments; n, individual mice.

**Figure 3.**
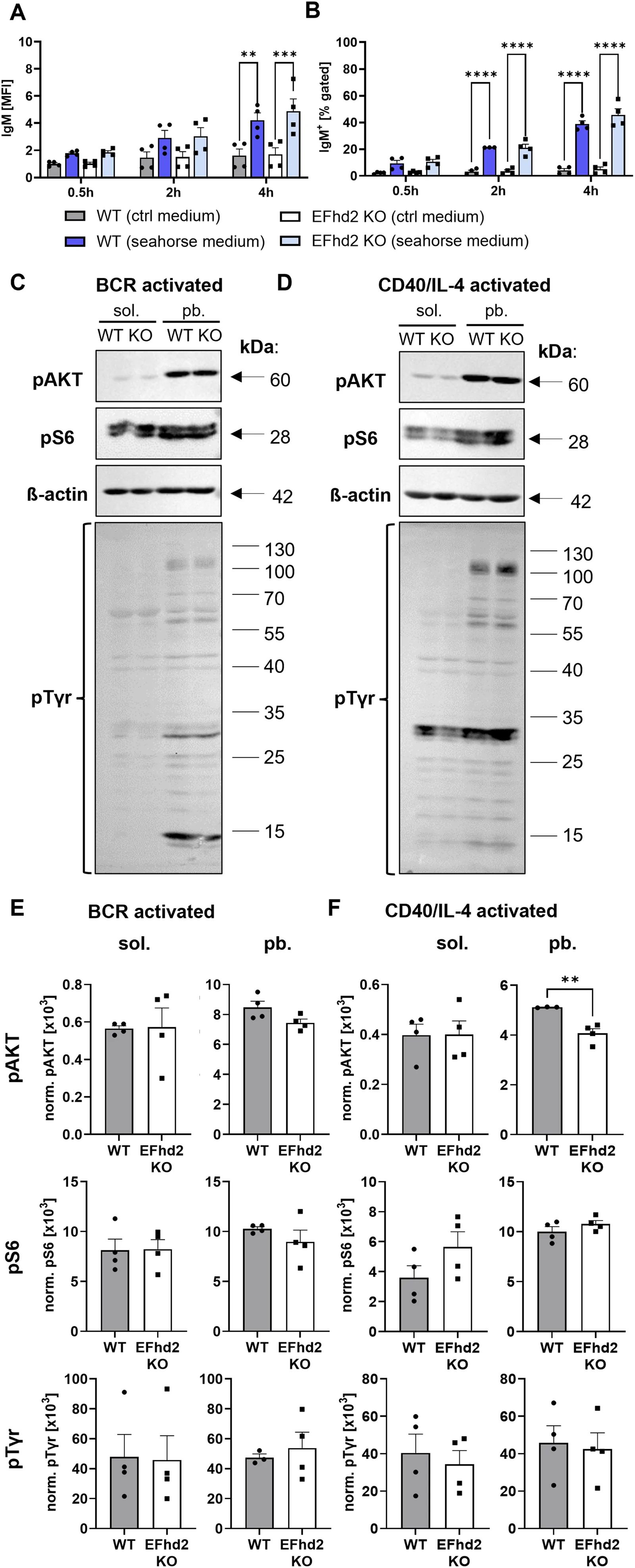
Normal BCR recycling and signaling in EFhd2KO B cells. **(A, B)** Splenic wild type (WT) and EFhd2 KO B cells were activated with anti-IgM Ab for 24 h. Cells were either maintained in stimulation medium (ctrl medium; ctrl) or transferred for the indicated time points into Seahorse medium lacking anti-IgM stimulation. Activated cells (CD19^high^, B220^high^ and CD86^+^) were stained with anti-IgM PE. **(A)** Mean fluorescence intensity (MFI) of surface IgM expression. **(B)** Frequencies of IgM positive (IgM^+^) B cells at the indicated time points. Data are presented as mean ± SEM; each dot represents one mouse; statistics: N = 2, n = 4, Two-way ANOVA (*P*-values <0.5. **P < 0.01, ***P < 0.002, ****P < 0.0004). **(C, D)** Splenic B cells from wild type (WT) and EFhd2 KO mice were stimulated for 24 h with anti-IgM Ab or anti-CD40/IL-4 (sol.) and then seeded on anti-BCR coated plates (plate bound, pb). Proteins were extracted, separated by SDS–PAGE, and transferred to nitrocellulose membranes. Membranes were probed with antibodies as indicated. **(C, D)** Representative immunoblots of WT and EFhd2 KO B cells after anti-IgM and CD40/IL-4 stimulation. **(E, F)** Quantification of pAKT, pS6 and pTγr normalized to actin. Data are presented as mean ± SEM; each dot represents one mouse; statistics: N = 1, n = 4; unpaired *t* test (*P*-values <0.5. **P < 0.01). N, number of experiments, n, individual mice.

Because activated EFhd2KO B cell cultures contain more cells with high mitochondrial mass we tested whether EFhd2 would impact mitochondrial respiratory activity. We continued to work with activated B cells because (a) non-activated B cells are metabolically rather inert (Caro-Maldonado et al, 2014), (b) sorted GC B cells would be too small in number and short in viability and (c) are heterogeneously imprinted by various signals. Hence, we analyzed 24 h soluble anti-IgM activated cells attached to Poly-D-Lysine (PDL) by extracellular flux analysis via a Seahorse Mito Stress Test (MST) (Fig 2). A MST first measures the basal oxygen consumption, followed by the assessment of ATP-linked respiration and proton leak after inhibition of ATP synthase with Oligomycin. Maximal respiratory capactiy is then determined by the addition of FCCP, which uncouples oxidative phosphorylation. Finally, non-mitochondrial respiration is measured following the sequential addition of Rotenone and Antimycin A, inhibiting Complex I and III, respectively. Spare capacity is defined as the difference between maximal and basal respiration (Fig 2A). The standard MST performed with 24 h soluble anti-IgM activated cells attached to PDL showed that EFhd2KO B cells display a tendency toward higher respiration, proton leak and spare respiratory capacity (SPRC) (Fig 2B, D, and E), which correlates with the higher proportion of cells containing more mitochondrial mass (Fig 1C). To simulate an immune synapse, we subsequently coated the Seahorse assay plates with anti-IgM antibodies, to which the cells attach for the duration of the Seahorse assay. Strinkingly, all respiratory parameters increased on anti-IgM antibodies in WT B cells, but not in EFhd2KO B cells (Fig 2C–E). This difference was not due to altered viability or cell cycle induction of the stimulated cells as assessed by Annexin V and DNA staining (Fig S1B–F), or limiting coating concentrations of anti-IgM antibodies (Fig S2A and B). Importantly, also after soluble anti-CD40/IL-4 stimulation, anti-IgM attached WT B cells up-regulate SPRC significantly more than EFhd2KO B cells (Fig 2F–H) although here, in contrast to BCR activation, EFhd2KO B cells also elevate SPRC to some extent (Fig 2I). Although we plated the cells separately on either anti-IgM or PDL-coated plates, we believe that the considerable number of experiments and independent preparations of B cells from different mice speak against a systematic error elicited by using plates coated with either PDL or anti-IgM antibodies. Moreover, there was no difference in CD19, B220, CD86, or IgM surface abundance (Fig S2C and D), confirming equal activation, and the IgM BCR recycled equally to the cell surface in the experimental conditions (Seahorse basal XF medium without anti-IgM stimulation, comparable to MST conditions) in WT and EFhd2KO B cells (Fig 3A and B). In addition, there was no clear reduction of Akt, S6 or total Tyrosine phosphorylation in any condition, except of a small decline of pAkt in CD40/IL-4 activated B cells attached on anti-IgM coated plates (Figs 3C–F and S2E). This observation speaks against prominent signaling defects in EFhd2KO B cells, in line with our previous notions (Brachs et al, 2014). Moreover, it demonstrates BCR signaling competence even in BCR activated B cells in our conditions. Together, these experiments reveal that (i) activated B cells increase mitochondrial respiration, in particular SPRC, upon anti-IgM coated surfaces even when BCR expression is low, and (ii) require EFhd2 for this process.

### B cells increase mitochondrial spare capacity on immobilized anti-IgM antibodies partially via the Actin cytoskeleton

**Figure 4.**
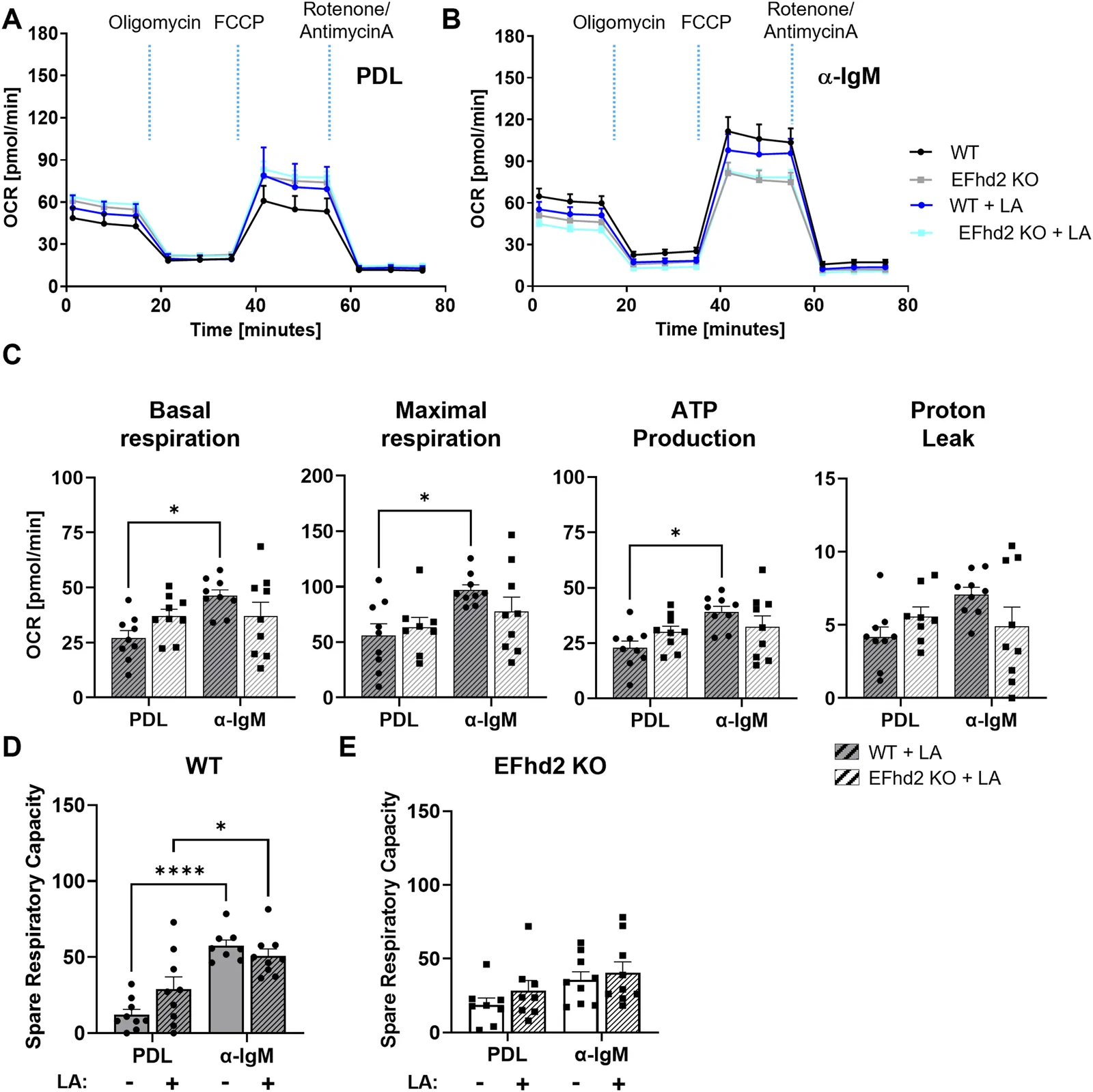
Latrunculin A decreases mitochondrial respiration, proton leak and spare capacity upon plate-bound IgM activation. **(A, B)** Representative extracellular flux analysis of 24 h anti-IgM Ab activated splenic B cells attached to poly-D-lysine (PDL) or anti-IgM Ab with or without Latrunculin A (LA) treatment. The basal oxygen consumption rate (OCR) was measured before and after injection of Oligomycin, FCCP and Rotenone + Antimycin A using the Seahorse Wave Mito Stress Test (MST) protocol. Symbols represent means of three mice, each of three to four replicate wells, mean ± SEM. **(C)** Calculated OCR of basal respiration, maximal respiration, ATP production and proton leak of 24 h anti-IgM activated B cells attached to PDL or anti-IgM Ab with LA treatment. Calculated spare respiratory capacity (SPRC) of activated WT (D) and EFhd2 KO (E) B cells treated with or without LA. **(C, D, E)** Data are presented as mean ± SEM; each dot represents one mouse; statistics: N = 6, n = 9; two-way ANOVA (*P*-values <0.5. *P < 0.05, ****P < 0.0004). N, number of experiments; n, individual mice.

EFhd2 stabilizes Actin filaments whereas activated EFhd2KO B cells exhibit a more dynamic Actin cytoskeleton (Reimer et al, 2020). Since immune synapse formation relies on Actin remodeling (Nowosad et al, 2016), EFhd2 is likely to play a role in this process. To test whether the Actin cytoskeleton is involved in connecting the BCR to mitochondrial respiration on anti-IgM coated surface, we treated WT and EFhd2KO B cells with the Actin-depolymerizing drug Latrunculin A (LA). LA exerted hardly any effect on WT B cells and on EFhd2KO B cells (Fig 4A and B). Although WT B cells were treated with LA, they still increased basal and maximal respiration as well as ATP production upon immune synapse formation (Fig 4B and C). The elevation of SPRC in WT B cells was, however, mildly impaired when compared with non-treated cells (Figs 4D and 2E). This was likely due to combined effects of a (non-significant) increase on PDL and a smaller increment on the anti-BCR-coated surface. SPRC in EFhd2KO B cells was unaffected (Fig 4E). This experiment shows that Actin dynamics has minor effects on the BCR-mitochondrial axis.

### B cells increase mitochondrial spare capacity on immobilized anti-IgM antibodies mainly via the tubulin cytoskeleton

**Figure 5.**
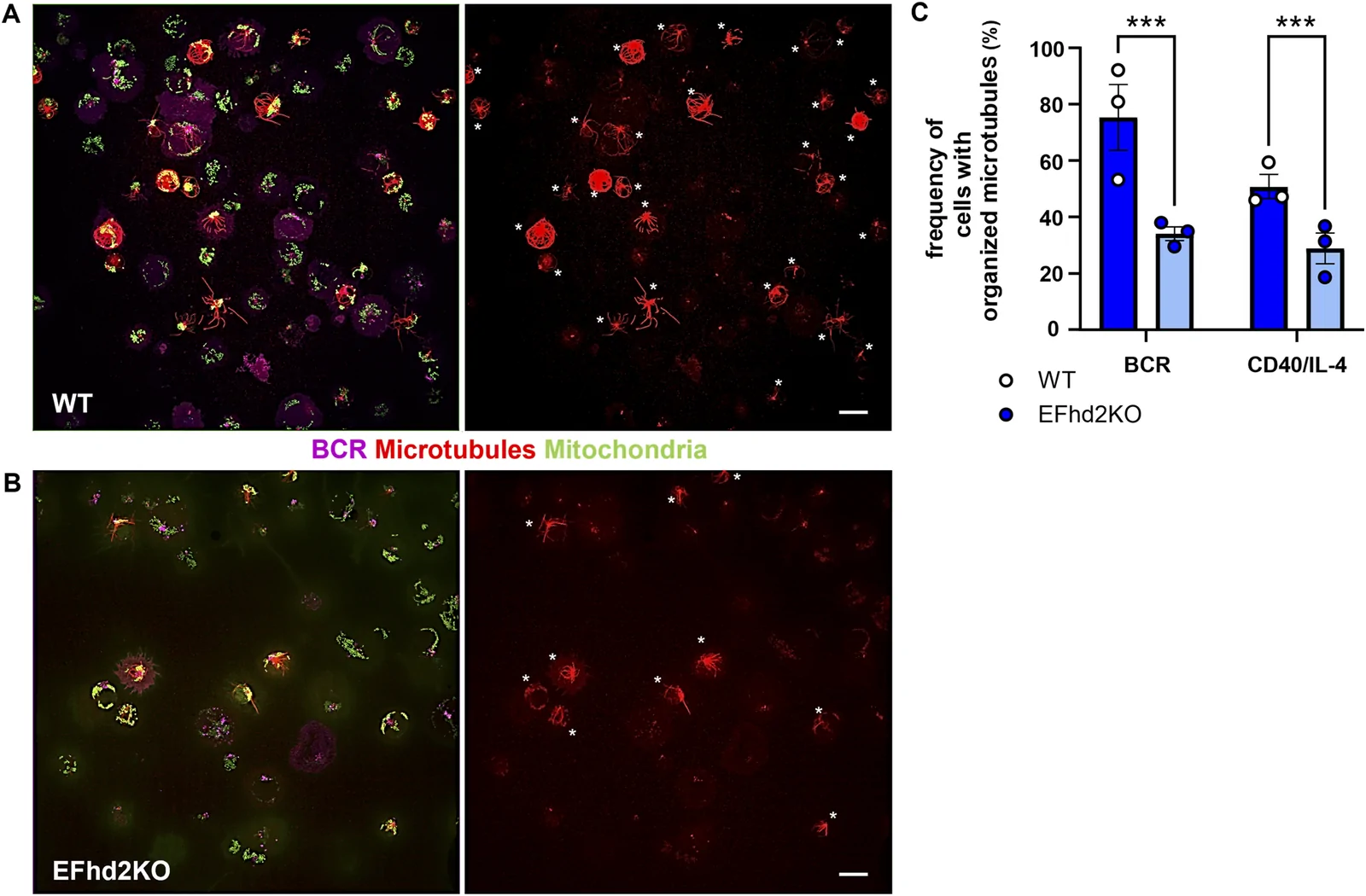
EFhd2 supports microtubule organization in anti-IgM-attached cells. **(A, B)** Splenic B cells from wild type (WT) (A) and EFhd2 KO mice (B) were stimulated with anti-IgM or anti-CD40/IL-4 for 24 h (data shown represent anti-CD40/IL-4 activated cells). Cells were labeled with Mitotracker Green, Tubulintracker DeepRed, and Alexa Fluor 594- conjugated anti-IgM Fab fragments, followed by attachment to plate-bound anti-IgM antibodies. Z-Stacks (0.26 mm slices, 21 layers) were acquired by live cell spinning disc super resolution microscopy. Images were deconvoluted and assembled as full stack (maximal projection). Cells with organized microtubules were counted (WT: 29/69; EFhd2KO: 10/37), marked with white stars. Scale bar, 10 μm. **(C)** Frequencies of cells with organized microtubules of anti-BCR or anti-CD40/IL-4 activated cells, see representative images in (A). Data are representative of three mice each with 3–4 technical replicates per mouse; data points depict the means of the technical replicates per mouse, representing 68–276 cells. Mann-Whitney U-Test (*P*-values <0.5. *** *P* < 0.001).

**Figure 6.**
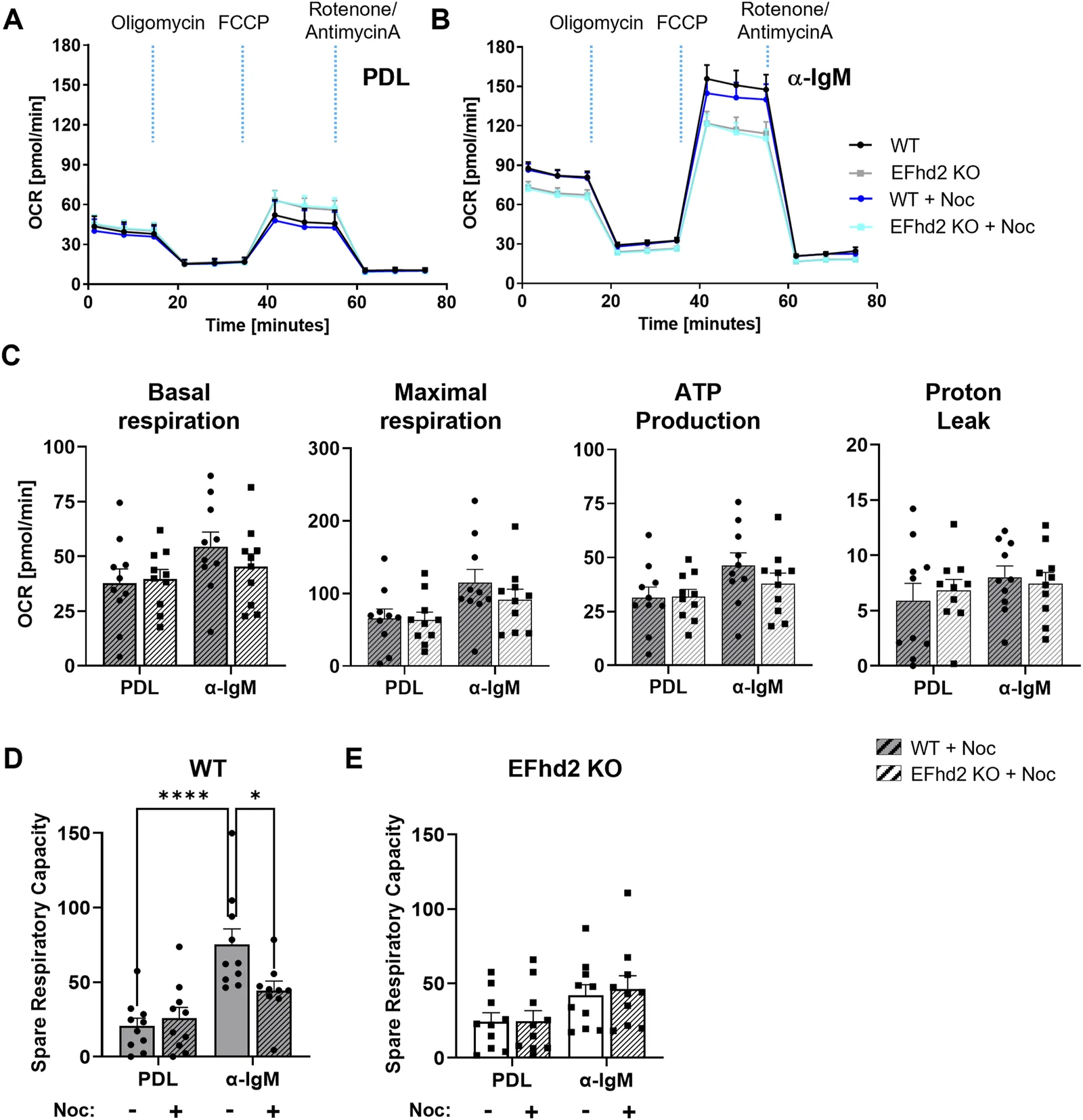
Microtubule destabilization decreases mitochondrial respiration, proton leak and spare capacity upon plate-bound IgM activation in WT B cells. **(A, B)** Representative extracellular flux analysis of 24 h anti-IgM Ab activated splenic B cells attached to poly-D-lysine (PDL) or anti-IgM Ab with or without Nocodazole (Noc) treatment. The basal oxygen consumption rate (OCR) was measured before and after injection of Oligomycin, FCCP and Rotenone + Antimycin A using the Seahorse Wave Mito Stress Test (MST) protocol. Symbols represent means of three mice, each of three to four replicate wells, mean ± SEM. **(C)** Calculated OCR of basal respiration, maximal respiration, ATP production and proton leak of 24 h anti-IgM activated B cells attached to PDL or anti-IgM Ab with Noc treatment. **(D, E)** Calculated spare respiratory capacity (SPRC) of activated WT (D) and EFhd2 KO (E) B cells treated with or without Noc. **(C, D, E)** Data are presented as mean ± SEM; each dot represents one mouse; statistics: N = 5, n = 10; two-way ANOVA (*P*-values <0.5. *P < 0.05, ****P < 0.0004). N: number of experiments, n: individual mice.

**Figure 7.**
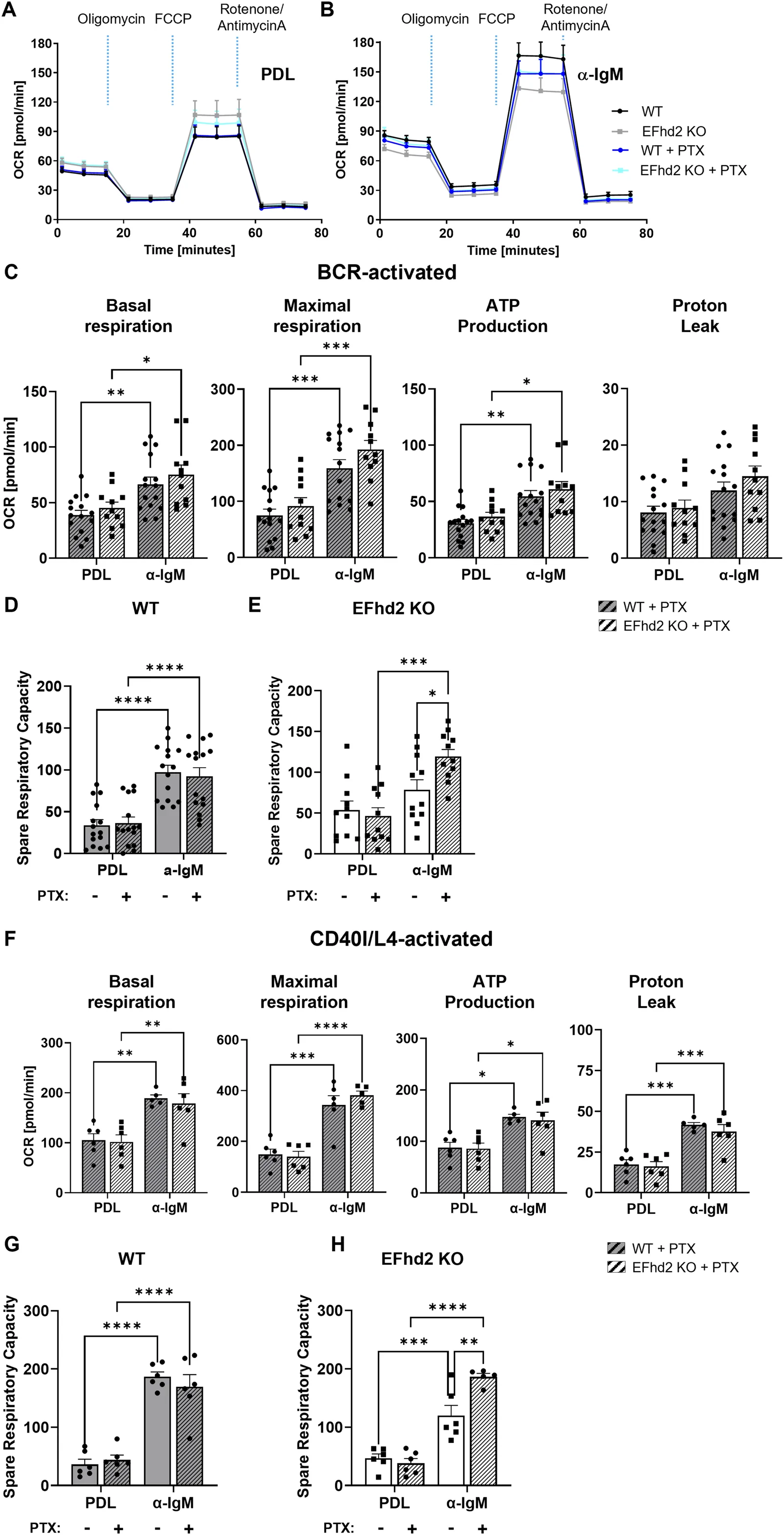
EFhd2-dependent microtubule stabilization drives respiration, ATP production, proton leak and spare capacity upon plate-bound IgM activation. **(A, B)** Representative extracellular flux analysis of 24 h anti-IgM Ab activated splenic B cells attached to poly-D-lysine (PDL) or anti-IgM Ab with or without Paclitaxel (PTX) treatment. The oxygen consumption rate (OCR) was measured before and after injection of Oligomycin, FCCP and Rotenone + Antimycin A using the Seahorse Wave Mito Stress Test (MST) protocol. Symbols represent means of two mice, each of three to four replicate wells, mean ± SEM. **(C)** Calculated OCR of basal respiration, maximal respiration, ATP production and proton leak. **(D, E)** Calculated spare respiratory capacity (SPRC) of activated WT and EFhd2 KO B cells treated with or without PTX. **(C, D, E)** Data are presented as mean ± SEM; each dot represents one mouse; statistics: N = 6, n = 11–15; two-way ANOVA (*P*-values <0.5. *P < 0.05, **P < 0.01, ***P < 0.002, ****P < 0.0004). **(F)** Calculated oxygen consumption rate (OCR) of basal respiration, maximal respiration, ATP production, proton leak and spare respiratory capacity of 24 h anti-CD40/IL-4 activated splenic WT and EFhd2 KO B cells attached to poly-D-lysine (PDL) or anti-IgM Ab cells after Paclitaxel (PTX) treatment. **(G, H)** Calculated spare respiratory capacity (SPRC) of anti-CD40/IL4-activated WT (B) and EFhd2 KO (C) B cells treated with or without PTX. Data are presented as mean ± SEM; each dot represents one mouse; statistics: N = 2, n = 6; two-way ANOVA (*P*-values <0.5. *P < 0.05, **P < 0.01, ***P < 0.002, ****P < 0.0004). N, number of experiments; n, individual mice.

**Figure S3.**
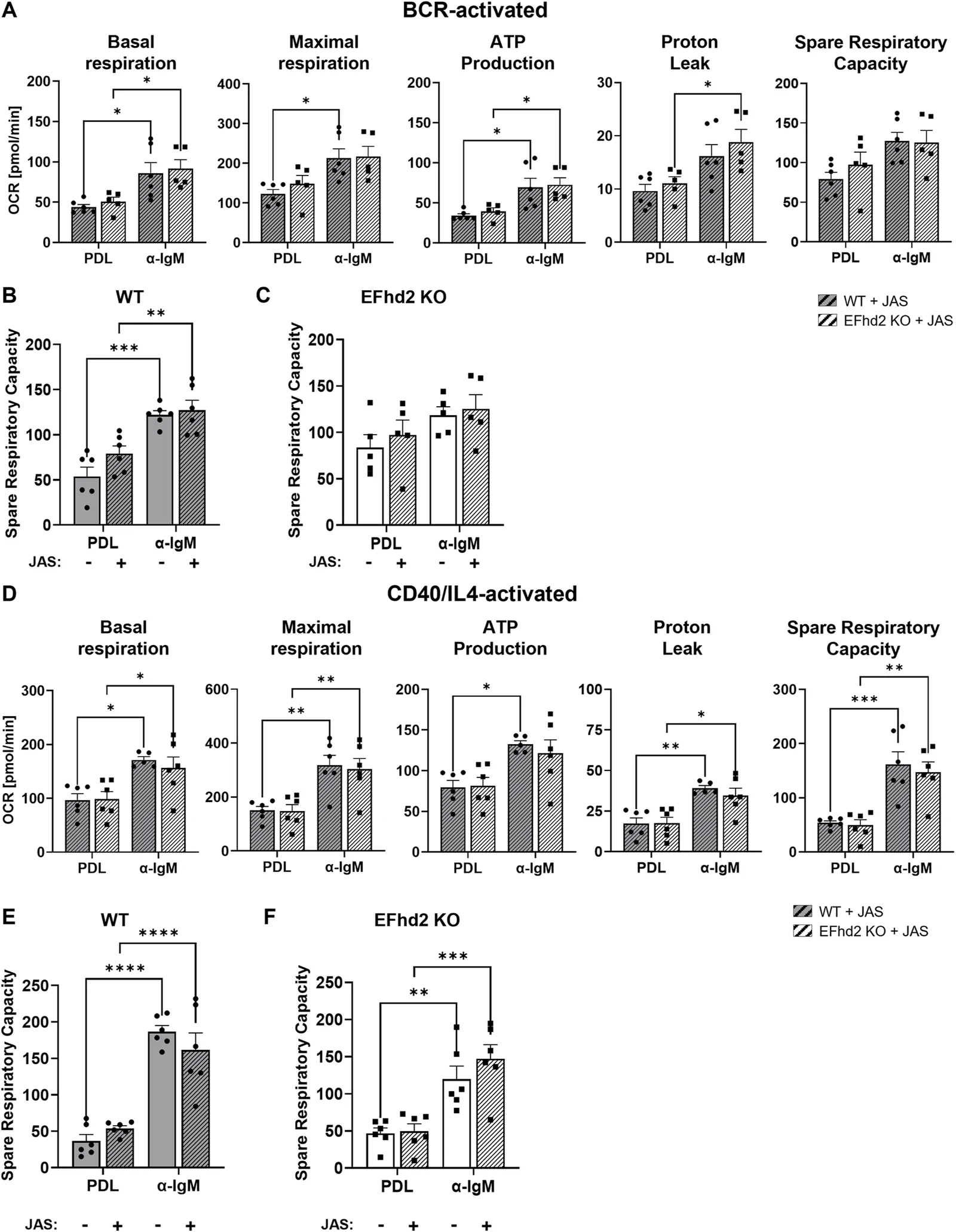
Jasplakinolide does not rescue mitochondrial spare capacity in BCR-attached EFhd2KO cells. **(A)** Calculated oxygen consumption rate (OCR) of basal respiration, maximal respiration, ATP production, proton leak and spare respiratory capacity of 24 h anti-IgM activated splenic WT and EFhd2 KO B cells attached to poly-D-lysine (PDL) or anti-IgM Ab cells after Jasplakinolide (JAS) treatment. **(B, C)** Calculated spare respiratory capacity (SPRC) of anti-IgM-activated WT (B) and EFhd2 KO (C) B cells treated with or without JAS. Data are presented as mean ± SEM; each dot represents one mouse; statistics: N = 2, n = 5–6; two-way ANOVA (*P*-values <0.5. *P < 0.05, **P < 0.01, ***P < 0.002). **(D)** Calculated oxygen consumption rate (OCR) of basal respiration, maximal respiration, ATP production, proton leak and spare respiratory capacity of 24 h anti-CD40 Ab + IL-4 activated splenic WT and EFhd2 KO B cells attached to poly-D-lysine (PDL) or anti-IgM Ab cells after Jasplakinolide (JAS) treatment. **(E, F)** Calculated spare respiratory capacity (SPRC) of CD40/IL4-activated WT (E) and EFhd2 KO (F) B cells treated with or without JAS. Data are presented as mean ± SEM; each dot represents one mouse; statistics: N = 2, n = 6; significance was analyzed using Two-way ANOVA (*P*-values <0.5. *P < 0.05, **P < 0.01, ***P < 0.002, ****P < 0.0004). N, number of experiments; n, individual mice.

**Figure S4.**
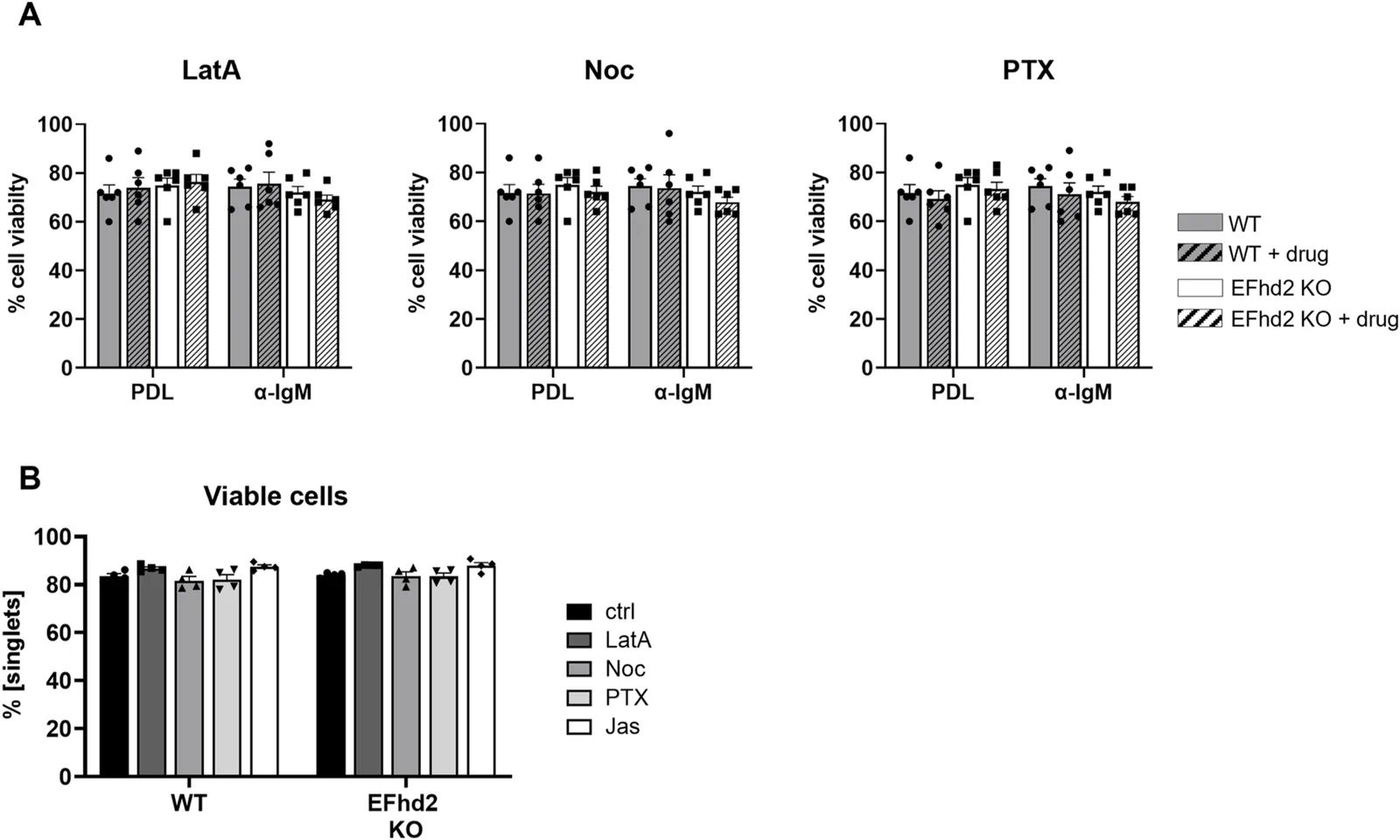
Similar viability of WT and EFhd2KO B cells after the mitochondrial stress test and in response to Latrunculin A, Nocodazole, Paclitaxel and Jasplakinolide. **(A)** Viability of splenic wild type (WT) and EFhd2 KO B cells activated with anti-IgM Ab for 24 h and treated with Latrunculin A (LatA), Nocodazole (Noc), or Paclitaxel (PTX). Cells were attached to poly-D-lysine (PDL) or anti-IgM–coated plates and analyzed for viability following the Seahorse Mito Stress Test by cell counter analysis. **(B)** Splenic B cells from WT and EFhd2 KO mice were stimulated with anti-IgM for 24 h and subsequently treated with LatA, Noc, PTX, or Jasplakinolide (Jas) for 60 min. Cell viability was assessed by flow cytometry using Cell Proliferation Dye eFluor 780 and ApoTracker Green and determined as double-negative cells (for gating strategy see Fig S1B). Data are presented as mean ± SEM; each dot represents one mouse; statistics: (A). N = 2, n = 6; (B). N = 1, n = 4, two-way ANOVA. No significant differences detected. N: number of experiments, n: individual mice.

Actin depolymerization did not completely suppress the elevation of SPRC on the BCR-coated surface in WT B cells. Besides the Actin system, microtubules are also involved in immune synapse organization but not many data in B cells are available (Kopf & Kiermaier, 2021). EFhd2 interferes with Kinesin-mediated transport along microtubules in vitro (Purohit et al, 2014). Moreover, EFhd2KO B cells appear to lose polarity during cell migration, a process that is coordinated by microtubule-dependent anterograde transport (Reimer et al, 2020; Kopf & Kiermaier, 2021). Mitochondria are anchored to the microtubule system via adaptor proteins, Kinesin, and Dynein (Jayashankar & Rafelski, 2014; Van Steenbergen et al, 2022; Huang et al, 2023). In our experimental setting, EFhd2KO B cells reorganized their microtubule system qualitatively less efficient when attached to the anti-IgM coated surface than WT B cells (Fig 5A–C), suggesting that EFhd2-mediated microtubule organization fosters SPRC. Therefore, activated EFhd2WT and KO B cells were attached to the anti-IgM coated surface in the presence of Nocodazole (Noc), which depolymerizes microtubules (De Brabander et al, 1977). In comparison to LA, Noc reduced the prominent increase of basal and maximal respiration, ATP production, proton leak and SPRC in anti-IgM attached WT B cells (Fig 6A–D). This suggested in fact a microtubule-dependent process although the efficacy of Noc on microtubule organization was not tested here formally. Strikingly, however, Noc did not exert any effect on EFhd2KO B cells (Fig 6E). This suggested that both, EFhd2 as well as a stable microtubule cytoskeleton, are essential for the BCR-mitochondrial axis. To challenge this notion, we chose the opposite approach and applied Paclitaxel (PTX) to stabilize microtubules (Schiff et al, 1979). PTX effectively elevated basal and maximal respiration, ATP production and proton leak in BCR as well as in anti-CD40/IL-4 stimulated EFhd2KO B cells attached to anti-IgM antibodies (Fig 7A–C, F, and G). Moreover, whereas PTX minimally modulated SPRC in anti-IgM attached WT B cells (Fig 7D and G), it fully restored SPRC of EFhd2KO B cells upon attachment to the anti-IgM coated surface (Fig 7E and H). Together, these data unequivocally demonstrate that heightened SPRC elicited by plate-bound IgM engagement depends on EFhd2-mediated microtubule stabilization. To test further for the specificity of microtubule stabilization, we treated the cells with Jasplakinolide, which stabilizes Actin filaments (Holzinger & Meindl, 1997). Actin stabilization with Jasplakinolide rescued mitochondrial function of BCR-attached EFhd2KO B cells to a lesser extent than Paclitaxel (Fig S3A–F), which is in agreement with the moderate effects of Actin destabilization by Latrunculin A (Fig 4). Noteworthy, none of the used Actin or Tubulin-modulating pharmaceuticals affected cellular viability (Fig S4A and B). We conclude that cytoskeletal stabilization restores SPRC of EFhd2KO B cells attached to an anti-IgM coated surface, with microtubules exerting a dominant influence.

### EFhd2KO B cells fail to organize mitochondria, microtubules and the BCR symmetrically in the synapse

**Figure S5.**
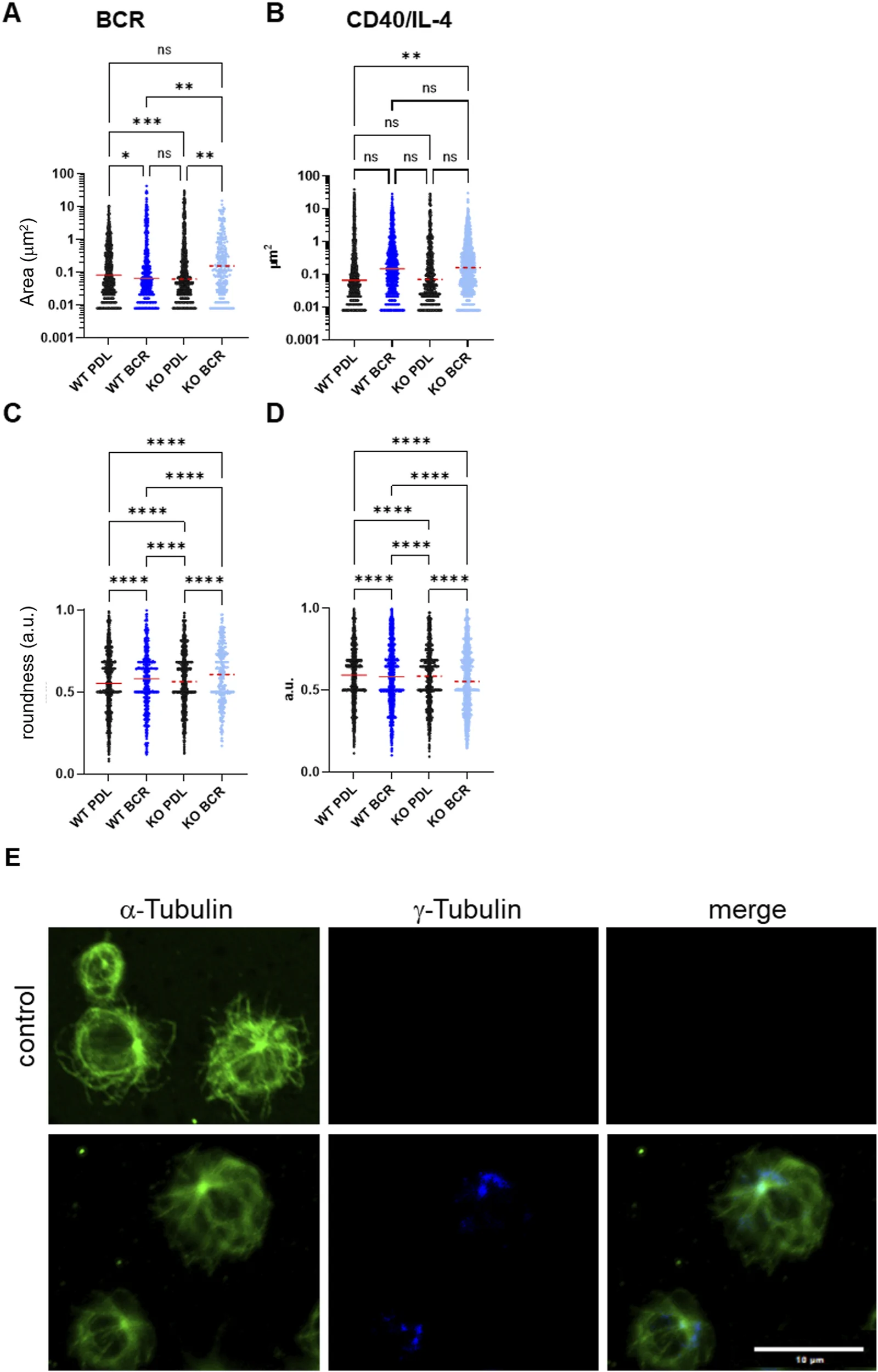
Mitochondrial size and shape, MTOC. **(A, B, C, D)** BCR or anti-CD40/IL-4 activated WT and EFhd2KO B cells were labeled with Mitotracker Green, Tubulin Tracker DeepRed and Alexa Fluor 594 conjugated anti-BCR Fab fragments. Cells were attached to Poly-D-Lysin (PDL) or anti-BCR antibodies in Seahorse medium. Z-stacks (260 nm) were recorded by super resolution confocal spinning disc live cell imaging. Mitochondrial fluorescence of a 1 μm stack in the central plane of the cells were analyzed for their size (A, B) or roundness (C, D). Data are compiled of three stimulations and represent 3 WT and 3 EFhd2KO mice (260–3,037 mitochondria). Two-way ANOVA (*P < 0.05, **P < 0.01, ***P < 0.002, ****P < 0.0004). **(E)** Anti-CD40/IL-4 activated WT B cells were attached to anti-IgM antibodies coated on glass slides, fixed with 4% PFA, permeabilized with 0.1% Triton X-100, stained with anti-α- and γ-Tubulin antibodies, mounted and analyzed by confocal microscopy. A Z-stack (8 μm) was recorded and superimposed. Control: no primary anti-γ Tubulin antibody. Scale bar, 10 μm.

**Figure 8.**
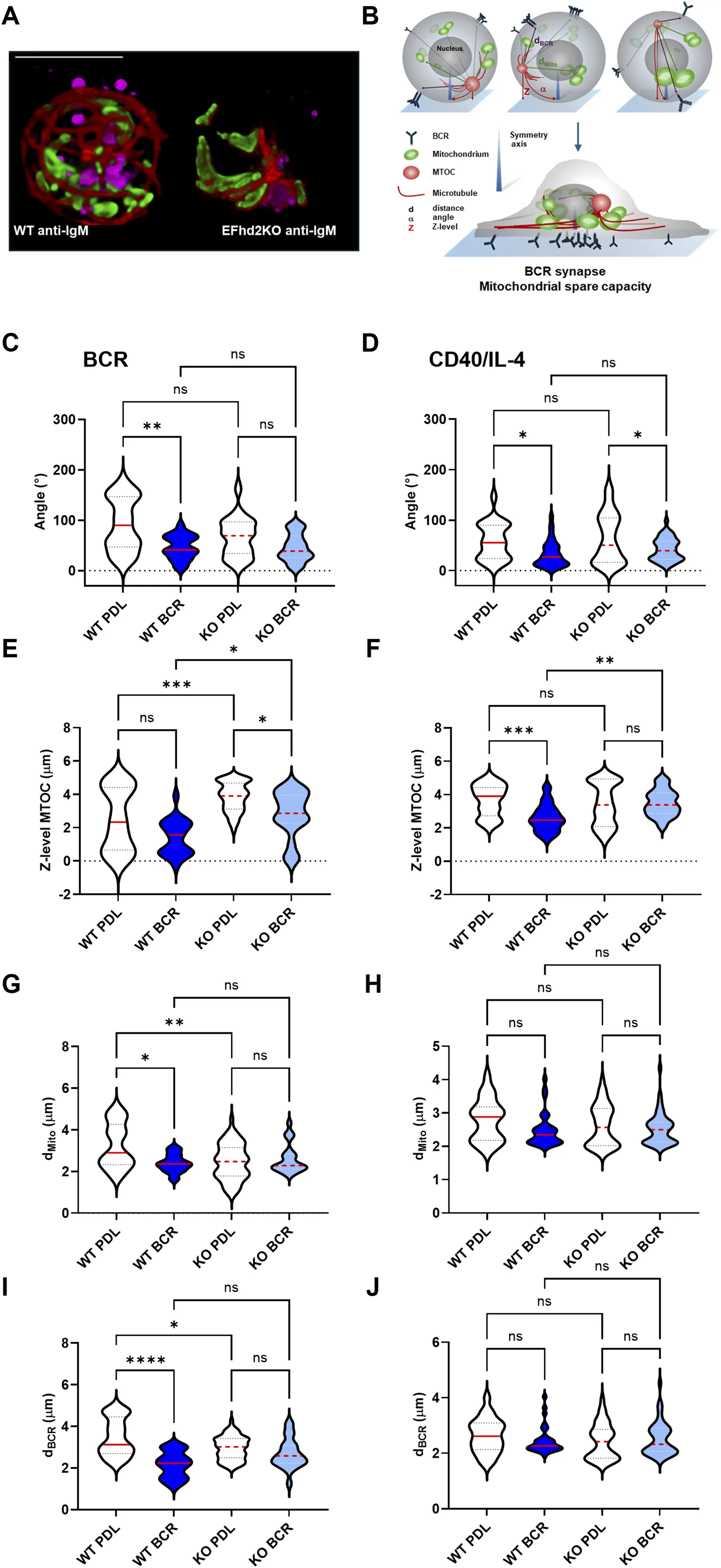
EFhd2 controls MTOC and mitochondria positioning .**(A)** Activated WT and EFhd2KO B cells were labeled with Mitotracker, Tubulin tracker and anti-BCR Fab fragments. Cells were attached to Poly-D-Lysin (PDL) or anti-IgM antibodies in Seahorse medium. Z-stacks (260 nm) were recorded by super resolution confocal spinning disc live cell imaging and 3D reconstructed (superimposed layers ranging 1–4 μm from the bottom of CD40/IL-4 activated cells shown, top view). Scale bar: 10 μm. **(B)** Model and analysis parameters (Z: distance in Z direction, MTOC – substrate; α: hypothetical angle required to center the MTOC to the cell-substrate symmetry axis; d_BCR_: weighted distance MTOC – BCR; d_Mito_: weighted distance MTOC – Mitochondria). PDL attached cells (upper) show a heterogeneous organization of the MTOC, mitochondria and the BCR. BCR-attached cells reorganize the BCR, the MTOC and mitochondria. **(C, D)** Z-level of the MTOC. **(E, F)**: angle. **(G, H)**: d_Mito._ **(I, J)**: d_BCR._ Data are compiled of three stimulations and represent 3 WT and 3 EFhd2KO mice with 34–71 cells, median values. Two-way ANOVA. ns, not significant, *P < 0.05, **P < 0.01, ***P < 0.002, ****P < 0.0004.

Owing to the microtubule-associated control of SPRC in our system, a correlation of EFhd2 with mitochondrial morphology seemed likely (Marchetti et al, 2020). Surprisingly, neither mitochondrial size nor their shape correlated with EFhd2-mediated spare capacity (Fig S5A–D). Therefore, we reasoned that— on top of macroscopic microtubule organization (Fig 5)— microtubule fine organization and the spatial arrangement of mitochondria via EFhd2d could be altered. Consequently, we visualized mitochondria, microtubules and the BCR in live cells with Mitotracker Green, Tubulin Tracker Deep Red and Alexa Fluor 594 labeled anti-IgM Fab fragments. This approach avoids the staining of intracellular B cell receptor reservoirs. B cells activated via the BCR or anti-CD40/IL-4 were adhered to PDL or anti-IgM coated glass slides and analyzed by super resolution spinning disc live cell microscopy. Representative 3D reconstructions of confocal Z-slices taken at distances ranging from 1 to 4 μm from the bottom reveal that WT B cells attached to IgM organize BCR clusters symmetrically in the vicinity of an aster-like microtubule network along which mitochondria are embedded (Fig 8A, top view of the cells). In contrast, the microtubule and mitochondrial networks in IgM-attached EFhd2KO B cells are displaced more asymmetrically (Fig 8A). In general, IgM-attached EFhd2KO B cells showed a more heterogeneous morphology. To quantify these impressions, we defined the microtubule organizing center (MTOC) as an aggregation of α-Tubulin, from which the Aster-like microtubule network spreads out, as revealed by adjacent γ-Tubulin staining (Fig S5E). We then measured the distance of the MTOC to the substrate-level of the cells on PDL and on anti-IgM antibodies (Z), the hypothetical angle (α) required to center the MTOC to the vertical symmetry axis of the cells, and the weighted distances (integration of distance and pixel intensity) of the BCR and mitochondria to the MTOC (d_BCR_ and d_Mito_; see illustration in Fig 8B) with a custom-made Fiji Plugin (Schmid, B. https://github.com/bene51/MTOC-receptor-analysis). BCR and CD40/IL-4 activated WT B cells attached to anti-IgM reduced the hypothetic angle needed to turn the cell and center the MTOC to the symmetry axis (Fig 8C and D) when compared with PDL attachment. CD40/IL-activated EFhd2KO B cells also reduced the angle, but to a lesser extent (Fig 8D; WT: −28°, KO: −12°). The distance of the MTOC to the substrate (Z-level) was lower in BCR and CD40/IL-4 activated WT B cells attached to anti-IgM antibodies when compared with EFhd2KO B cells (Fig 8E and F). The weighted distance of the mitochondria and the BCR to the MTOC was reduced upon anti-IgM attachment of BCR activated WT B cells (Fig 8G and I) but not EFhd2KO B cells. Similar reductions, albeit not statistically significant, were observed in CD40/IL-4 activated B cells (Fig 8H, WT: -0.6 μm, KO: −0.1 μm; Fig 8J, WT: −0.3 μm, KO: −0,1 μm). This is in line with the capability of CD40/IL-4 activated—but not BCR activated—EFhd2KO B cells to elicit some SPRC (Fig 2E and I). In sum, surface-coated anti-IgM antibodies elicit MTOC repositioning towards the BCR synapse, as well as BCR and mitochondrial proximity toward the MTOC. Overall, the combination of MTOC repositioning toward a lower substrate distance and toward the BCR (which itself re-localizes to the synapse) positions mitochondria in closer proximity to the B cell synapse. This correlates with the EFhd2-dependent induction of SPRC.

## Discussion

Referring to previous findings that B cell synapses facilitate B cell activation and expansion (Batista et al, 2001; Carrasco et al, 2004), we demonstrate here directly that B cell synapses are coupled to mitochondrial function. Thereby, we shed light on the question on how B cell synapses enable metabolic reprogramming (Carrasco, 2023). We show by real time and quantitative analysis that B cell synapses induced by immobilized anti-IgM Ab elevate basal and maximal mitochondrial respiration, proton leak, ATP production and in particular SPRC through EFhd2-mediated microtubule stabilization. This process involves spatial arrangement of the MTOC and mitochondria towards the center of the synapse. Spatial mitochondrial clustering has been shown to support local mitochondrial efficiency, i.e., SPRC, at neuronal synapses and myofibrils (“reservoir concept”) (Cserep et al, 2018; Rangaraju et al, 2019; Marchetti et al, 2020). In support of this interpretation, EFhd2 is a neuronal synaptic protein (Purohit et al, 2014), raising the possibility that mitochondrial function might also be altered in EFhd2-deficient neurons, causing premature cell death (Regensburger et al, 2018). Synapses are also specialized in Ca^2+^ handling and EFhd2 is a Ca^2+^ binding protein (Hagen et al, 2012). Ordered Ca^2+^ channeling in synapses may support mitochondrial function locally (Balaban, 2002; Feofilaktova et al, 2025). Regarding B cell biology, it is tempting to speculate that the achieved SPRC supports the resilience of activated B cells or LZ GC B cells to catabolic stress before the cells switch to anabolic pathways and clonal expansion after co-stimulation (Ersching et al, 2017; Long et al, 2022). Our findings synergize with other concepts such as signaling in explaining why repetitive synthetic or natural antigen arrays, such as viruses (Dintzis et al, 1976; Perotti & Perez, 2019), or immobilized antigen on (F)DC, elicit humoral immune responses of higher magnitude than soluble antigens or antigens of lower valency. A major implication of this work is that mitochondrial respiration in B cells is not only governed by nutrient availability, mtDNA abundance (Urbanczyk et al, 2022; Yazicioglu et al, 2023) and pO_2_, but also by cytoskeletal dynamics. This integration activates metabolic adaptation at the immune synapse, which is crucial for B cell survival, activation and antibody production. Previous data indicate that the BCR elicits antigen polarization and mitochondrial reprogramming via Protein kinase C β (Tsui et al, 2018). Here, we follow up on these data to show that BCR polarization is linked to mitochondrial condensation at the BCR synapse. This proposal aligns with the notion that mitochondrial transport is controlled by Kinesin and Dynein activity as well as Tubulin conformation (Van Steenbergen et al, 2022). The mitochondrial reorganization correlated predominantly with SPRC under our conditions. A high mitochondrial SPRC equips cells with a high adaptive capacity towards stress and relies on a well-organized network of fused mitochondria. Vice versa, a low SPRC correlates with distorted mitochondria and sensitizes cells to metabolic stress (Marchetti et al, 2020); however, mitochondrial size or shape did not correlate with SPRC in our hands. SPRC can also be attributed to a mildly increased proton leak (Marchetti et al, 2020). Indeed, proton leak was reduced in BCR and in CD40/IL-4 activated EFhd2KO B cells. With respect to synapse architecture, the induction of SPRC correlated best with the EFhd2-mediated MTOC relocation to the center and to the base of the synapse, suggesting that EFhd2 links the BCR to the MTOC. However, as to microtubules there is still a gap in understanding how exactly EFhd2 controls microtubule and mitochondrial dynamics in B cells. It is known that EFhd2 slows vesicle transport in primary mouse neurons and recombinant EFhd2 hinders Kinesin-mediated microtubule gliding (Purohit et al, 2014). This would indicate more Kinesin activity in BCR-attached EFhd2 B cells and a shifted equilibrium of minus/plus end transport of mitochondria, a hypothesis worth testing in the future. In the mouse hindbrain, EFhd2 interacts with proteins of the Actin, microfilament and Tubulin cytoskeleton, as well as with proteins involved in mitochondrial regulation (Soliman et al, 2021). In particular, there is an association between EFhd2, VPS13C (vacuolar protein sorting 13 homolog C) and Dynamin 1, which maintain mitochondrial membrane potential and mitochondrial fission in neurons, respectively (Soliman et al, 2021). Moreover, Dynamin 1 is a key mediator of Clathrin-dependent endocytosis. In line, in cancer cell lines EFhd2 functions as a cargo-specific adaptor in Clathrin- and Dynamin-independent endocytic pathways, facilitating the internalization and trafficking of various receptors, including adhesion receptors (Moreno-Layseca et al, 2021). However, here we show that EFhd2 does not regulate BCR recycling. Regarding signaling it has been revealed that the phosphatidylinositol-3-kinase-Akt-mTOR pathway guides metabolic reprogramming in B cells (Jellusova, 2020). Albeit phosphorylation of Akt was slightly compromised in BCR-attached EFhd2KO B cells, this reduction is unlikely to explain the absolute absence of elevated SPRC under these conditions, while mTOR activity in BCR or anti-CD40/IL-4 activated EFhd2KO B cells was unaltered. Overall, we propose here that the BCR communicates with mitochondria via the cytoskeleton, enabling B cells to discriminate antigenic nature. Along this line, EFhd2 appears to modulate T-cell activation differently upon soluble anti-CD3/CD28 or bead-bound anti-CD3/CD28 stimulation and is a component of the TCR synapse (Peled et al, 2018; Zhang et al, 2024). Owing to the complex interplay of signaling pathways and receptors on B cells we simplified the experimental setup, limiting the study to activating anti-BCR or anti-CD40 antibodies with IL-4 supplementation. This approach makes our data well comparable with other studies (Caro-Maldonado et al, 2014; Akkaya et al, 2018). BCR activation via anti-IgM antibodies for 24 h drives S-Phase entry; however, it does undoubtedly elicit apoptosis and downmodulates surface BCR abundance. Apoptosis, as well as low BCR expression, like in our BCR activated cells, are natural hallmarks of the GC environment (Paus et al, 2006; Chan et al, 2009; Kwak et al, 2018). In that regard, we believe that our data can be extrapolated to GC B cells or other B cells being repeatedly stimulated with antigen. Those also include self-reactive B cells: Polyvalent autoantigen complexes activate autoreactive B cells under physiological conditions and repeated encounter of autoantigen complexes leads to the production of affinity-matured autoreactive IgM (Amendt & Jumaa, 2022). Membrane-bound self-antigen elicits low IgM surface abundance and receptor editing or mitochondrial directed clonal deletion (Fang et al, 1998; Burnett et al, 2018). Thus, balancing mitochondrial fitness with intrinsic survival pathways in distinct B cell differentiation states influences the fate of B cells (Akkaya et al, 2018). As there was no difference in apoptosis or cell cycle entry we believe that apoptosis cannot explain the multiple metabolic and cell biologic differences observed between WT and EFhd2KO B cells. In addition, CD40/IL-4 stimulation, which is tolerated better by B cells, yielded similar results as BCR stimulation. Stimulating a B cell synapse via coating anti-IgM antibodies on glass surfaces and Seahorse reaction plates creates a rigid artificial antigen array. Antigen presentation on FDC may most closely resemble this model system as FDCs possess a rather stable and rigid surface when compared with other DCs (Nowosad et al, 2016). We did observe that EFhd2 supports B cell-FDC synapses in a B cell intrinsic manner in vivo and that EFhd2KO GC B cells vanish under competitive conditions (Reimer et al, 2020). Hence, we endorse the concept of EFhd2-mediated mitochondrial reorganization and fueling in the LZ that could support affinity maturation. Future experiments will show whether EFhd2-mediated mitochondrial organization has also relevance in other than B cells and identify cytoskeletal key players and receptors in this process.

## Materials and Methods

### Mice

The experiments were conducted using both female and male EFhd2-deficient mice (Brachs et al, 2014) on a C57BL/6 background. Wild-type littermates served as controls. All mice were housed under pathogen free conditions in the IVC at the Franz-Penzoldt-Center Erlangen (Germany). All experimental procedures were performed in accordance with ethical guidelines for animal experimentation and were approved by the government of Lower Franconia, Bavaria (Germany).

### Isolation of primary murine cells from spleen and mesenteric lymph nodes

Spleens and mesenteric lymph nodes were harvested and transferred into cold MACS buffer (Phosphate buffered saline [PBS], supplemented with 2% FCS). Tissues were gently passed through a 70 μm cell strainer (BD) using the plunger of a 5 ml syringe (BD). The resulting cell suspensions were pelleted by centrifugation at 400*g* for 5 min at 4°C. For spleen samples, erythrocytes were lysed upon resuspension in red blood cell-lysis buffer (150 mM NH_4_Cl, 10 mM KHCO_3_ and 1 mM EDTA) for 5 min at room temperature. The reaction was stopped by adding cold MACS buffer, followed by centrifugation at 400*g* for 5 min at 4°C. The final cell suspensions were filtered through a 30 μm mesh filter (Sysmex) and maintained in cold MACS buffer until further use.

### Purification and in vitro cultivation of primary murine B cells

Splenic B cells were enriched using the EasySep Mouse B cell isolation negative selection kit (EasySep #19854; StemCell Technologies) according to the manufacturerʼs instructions. Briefly, spleen cells were resuspended in MACS buffer, surface blocked with rat serum, and immunomagnetically enriched for untouched naive B cells. The purity of the isolated B cells was assessed by surface staining for CD19 and B220. Usually, an enrichment of >95% was achieved. Freshly isolated B cells were immediately cultured at a concentration of 2 × 10^6^ cells/ml in pre-warmed R10 medium (RPMI1640 supplemented with 10% fetal calf serum [FCS], 2 mM glutamate, 1 mM sodium pyruvate, 50 U/ml penicillin G, 50 μg/ml streptomycin and 50 μM ß-Mercaptoethanol) for 24–48 h at 37°C and 5% CO_2_. Cultures were supplemented with 10 μg/ml anti-IgM F(abʼ)_2_ Ab fragment (Jackson ImmunoResearch), 10 μg/ml anti-CD40 Ab (clone FGK45, Protein G purified from hybridoma supernatants) and 0.1 U/ml IL-4 (mouse IL-4, Miltenyi Biotec).

### Flow cytometry

0.5 × 10^6^–2 × 10^6^ cells were pelleted in FACS tubes (Micronic) at 300*g* for 5 min at 4°C and resuspended in 50 μl of unlabeled anti-FCGR3/CD16-FCGR2B/CD32 Ab (clone93; BioLegend) at 10 μg/ml in FACS-buffer (PBS, 2% fetal calf serum [FCS, 10270-106; Gibco Life Technologies], 0.05% sodium azide [K305.4; Carl Roth]) for 15 min on ice. Cells were washed once with FACS-buffer by centrifugation at 300*g* for 5 min at 4°C, resuspended in 50 μl FACS-buffer containing the respective fluorochrome-coupled Abs (anti-B220 APCFire750, 1:200, or BV421, 1:100, clone Ra3-6b2), anti-CD19 (clone 6D5, APCFire750, 1:400, BV421, 1:100, or BV510, 1:50), anti-CD86 (PECy7, clone A17199A, 1:200) (all Biolegend), goat anti-IgM (PE, Southern Biotech, 1:1,000), and incubated for 20 min on ice in the dark. For intracellular cell staining (anti-pS6 PE, clone cupk43k, eBioscience, 1:200), cells were permeabilized and fixed using the FoxP3/Transcription Factor Staining buffer set (eBioscience) according to the manufacturerʼs instructions. Cells were washed twice with FACS-buffer by centrifugation at 300*g* for 5 min at 4°C and data were acquired using a Gallios flow cytometer (Beckman Coulter). Analyses were performed using Kaluza version 1.3 and 2.1 (Beckman Coulter). Apoptosis was measured by incubating the cells with Via Dye eFluor780 (Thermo Fisher Scientific) and Apo Tracker Green after washing and incubation in the appropriate staining buffer (BioLegend). Cell cycle analysis was performed with labeling of unstained cells in 0.1% Triton X-100/0.1% sodium citrate/50 μg/ml propidium iodide (Nicoletti et al, 1991).

### Measurement of mitochondrial mass and membrane potential

Mitochondrial mass and membrane potential were assessed by staining cells with Mitotracker Green and DeepRed (Thermo Fisher Scientific), respectively, followed by flow cytometric analysis. For staining, 1 × 10^6^ – 4 × 10^6^ cells were resuspended in pre-warmed freshly made Mitotracker staining buffer (RPMI without supplements, 10 nM Mitotracker Green and 5 nM Mitotracker DeepRed, Thermo Fisher Scientific; 100 μl per 1 × 10^6^ cells). After incubation at 37°C and 5% CO_2_ for 30 min, cells were washed twice in RPMI and blocked with 50 μl unlabeled anti-CD16/32 Ab (10 μg/ml in RPMI) for 15 min on ice. Cells were then washed once by centrifugation at 400*g* for 5 min at 4°C, resuspended in 50 μl RPMI containing the appropriate fluorochrome-conjugated Abs and incubated for 20 min on ice in the dark. Afterward, cells were washed twice with PBS and immediately analyzed using a Gallios flow cytometer (Beckman Coulter). Data analysis was performed using Kaluza software version 2.1.

### Extracellular flux assay

Extracellular flux analysis was performed using a standardized protocol to measure the oxygen consumption rate (OCR) on a Seahorse XF 96 Analyzer (Agilent). Briefly, cartridges were calibrated overnight with 200 μl/well calibrant buffer (Agilent). Cell culture plates were coated with 100 μl/well Poly-D-Lysine (100 μg/ml in 1x Tris EDTA buffer) at room temperature (RT) for 1–2 h, or with anti-IgM F(abʼ)_2_ Ab fragment (as indicated, usually 10 μg/ml, in 1x TE buffer) over night at 4°C. Plates were then dried for 1 h at RT, and washed three times with ddH_2_O to remove unbound antibodies. Cells were resuspended in the respective Seahorse XF RPMI assay medium (supplemented with 1 mM pyruvate, 2 mM glutamine and 10 mM glucose for a Mito Stress Test), equilibrated for 45 min at 37°C in the absence or presence of Latrunculin A (1 μM), Nocodazole (0.1 μM), Paclitaxel (1 μM), Jasplakinolide (1 μm), and seeded at a density of 2.5 × 10^5^ per well in 180 μl. Measurements were performed at least in triplicates. To ensure proper attachment and even distribution, cells were briefly centrifuged. For plating on anti-IgM-coated plates and poly-D-Lysine, an additional incubation step for 0.5–4 h at 37°C and 5% CO_2_ was performed. Cells coated on anti-IgM antibodies or Poly-D-Lysine were treated equally regarding the different incubation times. The Mito Stress Test assays were performed according to the manufacturer’s instructions. Viability before and after the experiments was routinely assessed by propidium iodide exclusion in an automated cell counter (Nucleocounter NC-300, Chemometec).

### Spinning disc live cell confocal microscopy

Live cell microscopy was performed at 37°C and 5% CO_2_ using the Evident Super Resolution Spinning Disc microscope (LSM) with a 63× oil immersion objective. Glass slides (8 chambers, Ibidi) were coated with either anti-IgM F(abʼ)_2_ Ab fragment (10 μg/ml in PBS) or poly-D-Lysine (100 μg/ml in PBS) overnight at 4°C. Chambers were then blocked with 200 μl/well of 10 mg/ml fatty acid free BSA in PBS for 2 h at room temperature, followed by washing with pre-warmed PBS. Activated B cells were stained with different loading dyes (1 μM Mitotracker Green and 1 μM Tubulintracker DeepRed in RPMI) for 30 min at 37°C and 5% CO_2_ and with anti-IgM F(abʼ)_2_ Ab (AF 594, diluted 1:100 in RPMI, Jackson ImmunoResearch) for 20 min on ice in the dark. After staining, cells were washed twice with RPMI and seeded at a concentration of 5 × 10^5^ cells/ml in 200 μl/well in Seahorse XF medium. Fluorescence signals were detected immediately after seeding at appropriate wavelengths using standard filters (e.g., FITC, Cy5, DAPI, and Rhod). Signals and differential interference contrast (DIC) were recorded with an Evolve camera. Image acquisition, super resolution and deconvolution were performed using the Evident CellSense software. Fiji was used to further analyze the images; the weighted MTOC-mitochondrial and MTOC-BCR distances, the MTOC-Z distance and MTOC angle a were analyzed using a custom Fiji macro (Schmid, B. https://github.com/bene51/MTOC-receptor-analysis). Further image processing was carried out using Fiji software and the 3Dscript plugin (Schmid et al, 2019). Mitochondrial size and shape were analyzed by superimposing 5 Z-layers at the intermediate Z-level of the cells. Mitotracker fluorescence was converted to 8 bit, thresholds were defined and mitochondria were analyzed with the Fiji Analzye particles plugin. Perfectly round particles (=1) were excluded as they represent noise.

Cells with organized microtubules were identified macroscopically and qualitatively based on the presence of visible microtubule filaments.

### Confocal microscopy

8-well glass bottom polymer chambered coverslips (Roth or Thermo Fisher Scientific) were coated with anti-IgM F(abʼ)_2_ Ab fragment (10 μg/ml in PBS) overnight at 4°C. Activated B cells were stained seeded at a concentration of 1 × 10^6^ cells/ml on the wells. After 30 min, cells were fixed in freshly 4% paraformaldehyde, pH 7.0, at 37°C for 15 min, washed in PBS, permeabilized for 5 min with 0.1% Triton X-100 and blocked with 10% FCS in PBS for 30 min. Cells were stained with rabbit anti-γ-Tubulin antibodies (#T3559; Sigmal Aldrich, 1:100), followed by FITC-coupled mouse anti-Tubulin antibody and goat anti-rabbit antibodies conjugated to Alexa Fluor 647 (each 1:500). After washing in PBS, slides were mounted with Mowiol. Fluorescence signals were detected at appropriate wavelengths using standard filters with a 63× oil immersion objective in a Zeiss LSM700 microscope. Images were processed using Fiji software.

### Western blot

Soluble activated cells were kept in RPMI medium or plated on anti-IgM (10 μg/ml) coated tissue culture plates. Cells were washed in phosphate buffered saline (PBS) by centrifugation or on the tissue culture plate by adding PBS and aspiration. Cells were pelleted or scraped directly into buffer containing 1% Triton X-100, 150 mM NaCl, 25 mM Tris/HCl pH 7.5, 5 mM EDTA, 1 mM phenyl-methyl-sulfonyl-fluoride, 1 mM sodium vanadate, and 1 mM sodium fluoride on ice for 15 min. The lysate was cleared by centrifugation at 10,000*g* at 4°C for 15 min and supernatants were prepared for sodium dodecyl sulfate (SDS)-polyacrylamid gel electrophoresis (PAGE) according to standard procedures. After SDS–PAGE, proteins were transferred onto a nitrocellulose membrane, the membrane was washed with deionized water and stained with Ponceau S. The membrane was blocked with 5% skim milk powder in TBST (150 mM NaCl, 25 mM Tris/HCl pH 7.5, 0.1 Tween-20), followed by incubation with primary antibody diluted in TBST containing 3% bovine serum albumin (BSA), 0.1% NaN_3_ and four washes with TBST. Antibodies were mouse anti-Actin, A1978; Sigma-Aldrich (1 μg/ml), rabbit anti-pS6, #4858; Cell Signaling (1:1,000), mouse anti-phospho-tyrosine P-Tyr-100, #9411; Cell Signaling (0.2 μg/ml), mouse anti-pAkt Ser473, #4060; Cell Signaling) (1:1,000). The membrane was incubated with secondary horseradish peroxidase (HRP) conjugated antibody diluted (goat anti-rabbit IgG-HRP, 111-035-046, goat anti-mouse IgG-HRP, 115-035-008; Jackson Immunoresearch, each 1:10,000) in 5% skim milk powder in TBST. After washing, the membrane was incubated with enhanced chemiluminescence (ECL) before exposure to X-ray films. Quantification was performed by densitometry of scanned X-ray films with ImageJ 1.52p. Band intensities were measured and background of X-ray films was subtracted. The relative abundance of Actin bands in each lane was quantified and other bands were normalized to relative Actin band intensity (Table 1).

**Table 1.** Reagents and antibodies

Reagent or resource	Source	Identifier
Antibodies		
Anti-B220 (APCFire750, clone Ra3-6b2)	BioLegend	Cat# 103278
Anti-CD16/32,clone93	BioLegend	Cat# 101302
Anti-CD19 (APCFire750, clone 6D5)	BioLegend	Cat# 115558
Anti-CD19 (BV421, clone 6D5)	BioLegend	Cat# 103278
Anti-IgM F(abʼ)2 fragment (AF594)	Jackson ImmunoResearch	Cat# 115-587-020
Anti-pS6 (PE, clone cupk43k)	eBioscience	Cat# 12-9007-42
rabbit anti-γ-Tubulin	Sigma-Aldrich	Cat# T3559
Mouse anti-α-Tubulin Alexa488	Santa Cruz	Cat# sc-5286
Mouse anti-Actin	Sigma-Aldrich	Cat# A1978
Rabbit anti-pS6	Cell Signaling	Cat# 4858
Mouse anti-pTyr	Cell Signaling	Cat# 9411
Mouse anti-pAkt Ser473	Cell Signaling	Cat# 4060
goat anti-mouse IgG-HRP	Jackson Immunoresearch	Cat# 115-035-008
goat anti-rabbit IgG-HRP	Jackson Immunoresearch	Cat# 111-035-046
Tracker		
MitoTracker DeepRed	Thermo Fisher Scientific	Cat# M22426
MitoTracker Green	Thermo Fisher Scientific	Cat# M7514
TubulinTracker DeepRed	Thermo Fisher Scientific	Cat# T34077
Experimental Models: Organisms/Strains		
EFhd2 KO mice	Brachs et al (2014)	MGI#5707812,RRID:MGI:5707812
Chemicals, peptides, and recombinant proteins		
Anti-CD40, cloneFGK45,	Rolink et al (1996)	RRID:AB_2490239
Antimycin A	Sigma-Aldrich	Cat# A-8674
ß-Mercaptoethanol	Gibco - Thermo Fisher Scientific	Cat# 31350-010
Carbonyl cyanide-p-trifluoromethoxy-phenylhydrazone (FCCP)	Sigma-Aldrich	Cat# C2920
CellCount and viability solution 13	Chemometec	Cat# 910-3,013
Fetal bovine serum (FCS), heat-inactivated	Gibco	Cat# 10270-10
Glucose	Carl Roth	Cat# HN06.3
Goat anti-IgM AffiniPure F(ab’)2 µ chain specific	Jackson ImmunoResearch	Cat# 115-006-075
Interleukin-4, premium grade	Miltenyi Biotech	Cat#130-097-761
Latrunculin A	Sigma-Aldrich	Cat# 428026
L-glutamine	Gibco - Thermo Fisher Scientific	Cat# 25030-24
Nocodazole	Sigma-Aldrich	Cat# 487929
Oligomycin	Sigma-Aldrich	Cat# 75351
Paclitaxel	Sigma-Aldrich	Cat# T7191
Penicillin/streptomycin	Gibco - Thermo Fisher Scientific	Cat# 15140-122
Poly-D-Lysine	Sigma-Aldrich	Cat# P7280
Rotenone	Sigma-Aldrich	Cat# R8875
RPMI1640	Gibco - Thermo Fisher Scientific	Cat# 31870-25
Sodium pyruvate	Gibco - Thermo Fisher Scientific	Cat# 11360-039
XF Calibrant buffer	Agilent	Cat# 100840-000
XF RPMI Assay medium [pH 7,4]	Agilent	Cat# 103576-100
4-Hydroxy-3-nitrophenylacetyl (bovine serum albumin; BSA), fatty acid free	Sigma-Aldrich	Cat# A3803
Software and algorithms		
GraphPad Prism 9	GraphPad	https://www.graphpad.com/scientific-software/prism/
Kaluza	Beckman Coulter	https://www.beckman.de/flow-cytometry/software/kaluza
Seahorse Wave Desktop Software	Agilent	https://www.agilent.com/en/product/cell-analysis/real-time-cell-metabolic-analysis/xf-software/seahorse-wave-desktop-software-740897
Fiji	This report	https://github.com/bene51/MTOC-receptor-analysis
CellSense	Evident Scientific	https://evidentscientific.com/de/products/software/cellsens
Others		
EasySep Mouse B Cell Isolation Kit	StemCell	Cat# 19854
NucleoCounter NC-3000	ChemoMetec	N/A
μ slide 8-well polymer chambered coverslips	Ibidi Bioscience	Cat# 80821

## Supplementary Material

Reviewer comments

## Data Availability

Original data are available upon reasonable request. contact dirk.mielenz@fau.de.
