# Supplementary material for: EFhd2 regulates microtubule-dependent mitochondrial fitness at the B cell immune synapse
Source: Life Sci Alliance. 2026 Nov 4;10(1):e202603876. doi: 10.26508/lsa.202603876 (PMC13736178; doi:10.26508/lsa.202603876)
Supplement: Supplementary file 1 [file LSA-2026-03876_review_history.pdf]

EFhd2 Regulates Microtubule-Dependent Mitochondrial Fitness at the B Cell Immune Synapse

Leonie Weckwerth, Benjamin Schmid, Philipp Tripal, Ann-Kathrin Himmelreich, Jana Thomas, Katharina Pracht, Sophia Urbanczyk, Hannah Allnach, Anna Gottwald, George Schett, Ralph Palmisano, and Dirk Mielenz
DOI: https://doi.org/10.26508/lsa.202603876

Corresponding author(s): Dirk Mielenz, Friedrich-Alexander-Universität Erlangen-Nürnberg

Review Timeline:

Submission Date:	2026-08-10
Editorial Decision:	2026-09-24
Revision Received:	2026-10-02
Accepted:	2026-10-09

Scientific Editor: Sarita Hebbar

Transaction Report:

(Note: With the exception of the correction of typographical or spelling errors that could be a source of ambiguity, letters and reports are not edited. The original formatting of letters and referee reports may not be reflected in this compilation.)

Please note that the manuscript was previously reviewed at another journal and the reports were taken into account in the decision-making process at *Life Science Alliance.*

September 24, 2026

RE: Life Science Alliance Manuscript #LSA-2026-03876-T

Prof. Dirk Mielenz
Friedrich-Alexander-Universität Erlangen-Nürnberg
Department of Translational Immunology
Glueckstr. 6
Erlangen, Bavaria 91054
Germany

Dear Dr. Mielenz,

Thank you for submitting your revised manuscript entitled "EFhd2 Regulates Microtubule-Dependent Mitochondrial Fitness at the B Cell Immune Synapse".

We sought external input from an expert on the topic of your manuscript, and independent from the previous reviewers (as you had requested). Their comments are appended below.
As you will read, this expert acknowledges the potential value of this work whilst also raising conceptual and technical concerns. The lack of mechanistic insight into the relevance of mitochondria positioning within a cell is not a critera for consideration at Life Science Alliance. However, we agree with the expert that the revised manuscript still has some unresolved concerns in terms of inconsistencies and lack of appropriate validation.

In light of the potential interest in these findings, we would be happy to publish your paper in Life Science Alliance pending (1) resolution of the following points with minor edits, and (2) final revisions necessary to meet our formatting guidelines.

-address the expert's point on inconsistency in stimulation of cells with soluble antibodies or with immobilised antibodies and differences in recovery time. We suggest that you explicitly discuss this aspect as a limitation as commented on by the expert.
-clarify methods including (i) how cells with organised microtubules were defined and quantified, and (2) sequential treatment of different inhibitors.
-ensure conclusions drawn reflect the comparisons made (WT vs WT + LA) for figure 4A-C.
-acknowledge the lack of validation showing that nocodazole acts as expected when discussing the results of Figure 6.

Kindly provide a letter that includes a point by point response to these comments from the expert along with your revised manuscript with above requested changes highlighted.

MANUSCRIPT ORGANIZATION AND FORMATTING:

To avoid unnecessary delays in the acceptance and publication of your paper, please read the following information carefully. Full guidelines are available on our Instructions for Authors page, https://www.life-science-alliance.org/authors

Submission of a paper that does not conform to Life Science Alliance guidelines will delay the acceptance of your manuscript.

-Please define scale bar size for Figure 8A.
-Please provide concentrations of antibodies used in flow cytometry/immunostaining/immunoblotting.
-Please upload your main and supplementary figures as single files.
-Please add a Summary Blurb/Alternate Abstract in our system.
-Please add a Category for your manuscript in our system.
-Please add the X and Bluesky handles of your host institute/organization, as well as your own, and/or one of the authors, in our system.
-Please remove the number of words from the abstract.
-It is recommended to exclude figures from the manuscript text and upload them separately.
-Please rename "Disclosure and competing interests statement" to "Conflict of Interest Statement".
-Please consult our manuscript preparation guidelines https://www.life-science-alliance.org/manuscript-prep and make sure your manuscript sections are in the correct order.
-Please use the [10 author names et al.] format in your references (i.e., limit the author names to the first 10).
-Please add your main and supplementary figure legends to the main manuscript text after the references section and remove them from figures.
-We encourage you to revise the figure legend for figure S1 such that the figure panels are introduced in alphabetical order.
-The "Data Availability" section should be placed after the Materials & Methods section. Please consult our guidelines at

https://www.life-science-alliance.org/manuscript-prep#format.
-Please add an Author Contributions section to your main manuscript text and in our system.
-Please add callouts for Figures 5A-C; S1G and S3A-F to your main manuscript text.
-Kindly remove the list of Abbreviations from the title page.
-Please be sure that the authorship listing and order is correct.

We encourage our authors to provide original source data, particularly uncropped/-processed electrophoretic blots and spreadsheets for the main figures of the manuscript. If you would like to add source data, we would welcome one PDF/Excel-file per figure for this information. These files will be linked as supplementary "Source Data" files.

It is Life Science Alliance policy that if requested, original data images must be made available to the editors. Failure to provide original images upon request will result in unavoidable delays in publication. Please ensure that you have access to all original data images prior to final submission.

If you are planning a press release on your work, please inform us immediately to allow informing our production team and scheduling a release date.

We welcome submissions of potential cover images for the issue of LSA in which your work would appear. If you have high quality images associated with this work, please feel free to email these, with a caption, to the journal office.

LSA encourages authors to provide a 30-60 second video where the study is briefly explained. These videos will be appear embedded with the manuscript online at Life Science Alliance, and on social media to promote the published paper and authors (for examples, see https://docs.google.com/document/d/1-UWCfbE4pGcDdcgzcmiuJl2XMBJnxKYeqRvLLrLSo8s/edit?usp=sharing). Corresponding or first-authors are welcome to submit the video. Please submit only one video per manuscript. The video can be emailed to contact@life-science-alliance.org

FINAL FILES:

To upload the final version of your manuscript, please log in to your account: https://lsa.msubmit.net/cgi-bin/main.plex
You will be guided to complete the submission of your revised manuscript and to fill in all necessary information. Please get in touch in case you do not know or remember your login name.

The following items are required for acceptance.

-- An editable version of the final text (.DOC or .DOCX) is needed for copyediting (no PDFs).

-- High-resolution figure, supplementary figure and video files uploaded as individual files: See our detailed guidelines for preparing your production-ready images, https://www.life-science-alliance.org/authors

-- Summary blurb (enter in submission system): A short text summarizing in a single sentence the study (max. 200 characters including spaces). This text is used in conjunction with the titles of papers, hence should be informative and complementary to the title. It should describe the context and significance of the findings for a general readership; it should be written in the present tense and refer to the work in the third person. Author names should not be mentioned.

The license to publish form must be signed before your manuscript can be sent to production. A link to the license to publish form will be available to the corresponding author only. Please take a moment to check your funder requirements.

Reviews, decision letters, and point-by-point responses associated with peer-review at Life Science Alliance will be published, alongside the manuscript. If you do want to opt out of having the reviewer reports and your point-by-point responses displayed, please let us know immediately.

Thank you for your attention to these final processing requirements. Please revise and format the manuscript and upload materials as soon as you are able.

Thank you for this interesting contribution to the literature. We look forward to publishing your paper in Life Science Alliance.

Sincerely,

Sarita Hebbar, PhD
Scientific Editor
Life Science Alliance
http://www.lsajournal.org

EXPERT'S COMMENTS

"To briefly sum up my assessement of the study- the findings are quite interesting and potentially valuable for the immunological community. However, in my opinion, the technical aspects of the work are not sufficiently robust to support publication on the basis of minor revisions alone.

Weckwerth et al. demonstrate in their study, that IgM stimulated EFhd2 deficient B cells show slightly increased oxygen consumption when compared with WT cells, but fail to further increase mitochondrial activity when restimulated by immobilised IgM. The authors use different chemical compounds to assess the role of the cytoskeleton in this phenomenon.
Overall, the initial findings are quite interesting and highlight that EFhd2 governs mitochondrial activity in a different manner depending on whether the cells were stimulated with soluble antibodies or with immobilised antibodies. This is certainly a novel and interesting finding and draws attention to a more subtle and context dependent mode of mitochondrial regulation.
However my enthusiasm for the study is limited by a few shortcomings.
In my opinion, the presentation of the results is not always sufficiently clear. At several points, the reasoning is difficult to follow, key methodological details are lacking, and there are instances where the text does not appear to be fully consistent with the data presented.
More importantly the study relies on a limited set of experimental approaches which make it vulnerable to possible technical issues and overinterpretation of results. Lastly an important mechanistic insight is missing- why is the positioning of the mitochondria within the cell crucial in order to support overall functionality? The restimulation of the cells with immobilised IgM occurs over a rather short period of time, yet the effects on overall oxygen consumption/spare respiratory capacity are quite large. How is this achieved by the repositioning of the mitochondria?

A more detailed description of some of the concerns:

I find the whole setup of the experiments difficult for interpretation. Cells were prestimulated with anti-IgM and then seeded on poly-D-lysine treated plates and anti-IgM coated plates. From the material and methods part it seems like these two treatments were not compared side-by side (by e.g. coating one half of the plate with one reagent and the other one with a different reagent) but measured independently. The authors write: "For plating on anti-IgM-coated plates, an additional incubation step for 0.5-4 hours at 37{degree sign}C and 5% CO_2 was performed." Thus cells on anti-IgM coated plates seem to have been given a much longer recovery time and the direct comparison does not seem to be fair. Also since it appears that the cells from the anti-IgM coated plate are receiving anti-IgM signals trhoughout the experiment and the cells on the poly-L-lysine plate have lost their stimulus for the duration of the experiment this again makes it difficult to compare these two setups.

In Fig.1A the authors use MitotrackerGreen and MitotrackerDeepRed and define cells with "small functional", "large functional" and "dysfunctional mitochondria". The interpretation of these results in ambiguous in my opinion. No control is provided to show that the staining works as expected (e.g. FCCP treatment to increase the proportion of dysfunctional mitochondria). Also it is unclear to me whether there are any functional features associated with an increase of "large functional" mitochondria. Why would cells that were not activated have more "large functional" mitochondria than activated cells? Was the cell size considered when interpreting this results?

In fig.4 the authors say: "While LA reduced maximal respiration in WT B cells, there was no significant effect in EFhd2KO B cells (Figure 4A, B)."
To me it looks like latrinculin increases maximal respiration in the PDL setup (blue WT+LA is higher than black WT). Moreover it would be useful to provide statistics for this statement (there are statistics for the spare respiratory capacity after LA treatment in fig.D,E, but the sentence in the text comments on maximal respiration and only references A,B)

In fig.5 it would be important to better describe the experiment and analysis. How were cells with "organised microtubules" defined? The example experiment should show which cells were counted in general and which were identified as cells with "organised microtubules". Also, for fig.5C the authors should define their scale better. Does "0.8" mean that 80% of the cells were cells with organised microtubules?

For fig.6 the authors write: In comparison to LA, Nocodazole reduced the prominent increase of basal and maximal respiration, ATP production, proton leak and SPRC in anti-IgM attached WT B cells. Again, looking at fig.6B nocodazole seems to have a very minor effect on the treated cells (the blue line is barely bellow the black line). A control showing that nocodazole acts as expected is missing.

The description of experiments using different inhibitors is to me unclear.
In the material and method section the authors write:
"Cells were treated sequentially with Latrunculin A (1µM), Nocodazole (0.1µM), Paclitaxel (1µM), Jasplakinolide (1mm) or left

untreated, and then starved for 45min at 37{degree sign}C in a non-CO2 incubator."
What do the authors mean by "sequentially"? Did one set of cells experience all the treatments one after another? Why were the cells starved?"

Point to point reply

Reviewer

I find the whole setup of the experiments difficult for interpretation. Cells were prestimulated with anti-IgM and then seeded on poly-D-lysine treated plates and anti-IgM coated plates. From the material and methods part it seems like these two treatments were not compared side-by side (by e.g. coating one half of the plate with one reagent and the other one with a different reagent) but measured independently. The authors write: "For plating on anti-IgM-coated plates, an additional incubation step for 0.5-4 hours at 37{degree sign}C and 5% CO_2 was performed." Thus cells on anti-IgM coated plates seem to have been given a much longer recovery time and the direct comparison does not seem to be fair. Also since it appears that the cells from the anti-IgM coated plate are receiving anti-IgM signals trhoughout the experiment and the cells on the poly-L-lysine plate have lost their stimulus for the duration of the experiment this again makes it difficult to compare these two setups.

Answer: We believe that the considerable number of experiments and independent preparations of B cells from different mice speak against a systematic error elicited by using plates coated with either Poly-D-Lysine or anti-IgM antibodies. We appreciate the suggestion of shared coating of the plates with both Poly-D-Lysine and anti-IgM antibodies, which did unfortunately not come into our minds previously. To make this clear (we added a clear description in the Materials & Methods part): cells pre-stimulated with anti-IgM did NOT receive continuous treatment during the incubation on the Seahorse Plate or for other experiments. In line, prestimulation with anti CD40/IL-4 also showed a difference between WT and EFhd2KO B cells. Cells coated on anti-IgM antibodies or Poly-D-Lysine were treated equally regarding the different incubation times.

In Fig.1A the authors use MitotrackerGreen and MitotrackerDeepRed and define cells with "small functional", "large functional" and "dysfunctional mitochondria". The interpretation of these results in ambiguous in my opinion. No control is provided to show that the staining works as expected (e.g. FCCP treatment to increase the proportion of dysfunctional mitochondria). Also it is unclear to me whether there are any functional features associated with an increase of "large functional" mitochondria. Why would cells that were not activated have more "large functional" mitochondria than activated cells? Was the cell size considered when interpreting this results?

Answer: For internal control we did use FCCP, which is a standard method in our lab to control for the measurement of mitochondrial membrane potential by Tetramethyrhodamine (see for instance Stein et al., Cell death and differentiation, 2017; Urbanczyk et al., Cell reports, 2022). We tested beforehand whether Mitotracker DeepRed is sensitive towards FCCP, which it is, while Mitotracker Green is not (see

snippet inserted below, taken from the Dissertation of the first author, Dr. Leonie Weckwerth):

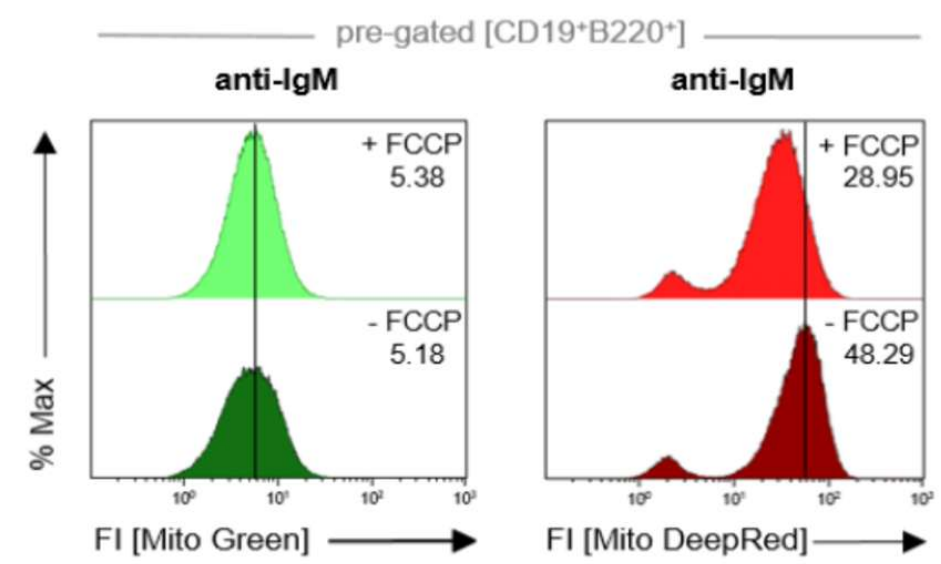

Sensitivity of Mitotracker DeepRed staining to inner mitochondrial membrane depolarization. Murine splenic B cells were activated with anti-IgM antibodies for 24h, the left untreated or treated with FCCP and stained with Mitotracker Green and Mitotracker DeepRed. Numbers indicate MFI.

The WT and EFhd2KO B cells were actually activated for the analysis in Figure 1, namely exactly to the same extent as can be seen in Figure S1 and S2. Therefore, cell size was not different. Of course, cell size does in principle matter, as we found out comparing large and small pre B cells previously (Stein et al., Cell death and differentiation, 2017). However, here this was not relevant. We based our combined Miotracker Green and Mitotracker Deep Red analysis on a previous report, which we quote now in the manuscript (Jang et al., Nat. Communications, 2015). Overall, our experiments do reveal clear differences between WT and EFhd2KO B cells. We appreciate that the reviewer noted that we described cells with high mitochondrial mass as “large mitochondria”, which is of course wrong. We identify now correctly populations with high or low mitochondrial mass and corrected this in the figure. Why EFhd2KO cell cultures contain a larger population of cells with high mitochondrial mass (we do not speak of larger mitochondria anymore) is admittedly still unclear, but may point to reduced mitochondrial turnover.

In fig.4 the authors say: "While LA reduced maximal respiration in WT B cells, there was no significant effect in EFhd2KO B cells (Figure 4A, B)." To me it looks like latrinculin increases maximal respiration in the PDL setup (blue WT+LA is higher than black WT). Moreover it would be useful to provide statistics for this statement (there are statistics for the spare respiratory capacity after LA treatment in fig.D,E, but the sentence in the text comments on maximal respiration and only references A,B).

Answer: We completely agree with this reviewer, we had the same impression and actually, Latrunculin A can mimick – or bypass – BCR signaling by enhancing the mobility of Actin cytoskeleton-restrained BCRs. We did statistics for all comparisons by 2-way ANOVA, as for all experiments, comparing all measurements in one graph with one another; however, there simply is no significant effect of Latrunculin A on PDL-attached WT B cells, even if we wished for. In this manuscript, we considered effects to be true when statistical significance was reached.

In fig.5 it would be important to better describe the experiment and analysis. How were cells with "organised microtubules" defined? The example experiment should show

which cells were counted in general and which were identified as cells with "organised microtubules". Also, for fig.5C the authors should define their scale better. Does "0.8" mean that 80% of the cells were cells with organised microtubules?

Answer: We marked the cells qualitatively assesed by us for organized microtubules in the example images. Frequencies can be depicted by different scales; we switched now to percentual representation.

For fig.6 the authors write: In comparison to LA, Nocodazole reduced the prominent increase of basal and maximal respiration, ATP production, proton leak and SPRC in anti-IgM attached WT B cells. Again, looking at fig.6B nocodazole seems to have a very minor effect on the treated cells (the blue line is barely bellow the black line). A control showing that nocodazole acts as expected is missing.

Answer: The reviewer is correct that in this particular example shown, the effect of Nocodazol is not very strong. It may be appreciated that throughout the paper we did quite a number of independent experiments, each including several independent B cell isolations from individual mice. This is what the statistics is based on. Nocodazole treatment is a standard condition in our experimental setups, effectively halting cells in G2 in the concentrations that we used. Overall, Nocodazol has clear effects on the mitochondrial activity of activated WT B cells under synapse-inducing conditions.

The description of experiments using different inhibitors is to me unclear.
In the material and method section the authors write:
"Cells were treated sequentially with Latrunculin A (1µM), Nocodazole (0.1µM), Paclitaxel (1µM), Jasplakinolide (1mm) or left untreated, and then starved for 45min at 37°C in a non-CO2 incubator."
What do the authors mean by "sequentially"? Did one set of cells experience all the treatments one after another? Why were the cells starved?"

Answer: We apologize for the wording. The cells were not treated sequentially; they were left untreated or treated with one inhibitor selectively. We removed the word “sequentially”. Starving refers to the incubation of the cells in the standard basal Seahorse XF medium, which does not contain foetal calf serum and which is a standard procedure for the Seahorse assay. We improved the experimental description.

October 9, 2026

RE: Life Science Alliance Manuscript #LSA-2026-03876-TR

Prof. Dirk Mielenz
Friedrich-Alexander-Universität Erlangen-Nürnberg
Department of Translational Immunology
Glueckstr. 6
Erlangen, Bavaria 91054
Germany

Dear Dr. Mielenz,

Thank you for submitting your Research Article entitled "EFhd2 Regulates Microtubule-Dependent Mitochondrial Fitness at the B Cell Immune Synapse". It is a pleasure to let you know that your manuscript is now accepted for publication in Life Science Alliance. Congratulations on this interesting work.

The final published version of your manuscript will be deposited by us to PubMed Central upon publication.

Your manuscript will now progress through copyediting and proofing. At the proofing stage, kindly include your explanation, "Cells coated on anti-IgM antibodies or Poly-D-Lysine were treated equally regarding the different incubation times" in the methods section (as per your response to the expert's first comment).

It is journal policy that authors provide original data upon request.

Your article will publish open access upon publication under a CC-BY license.

Reviews, decision letters, and point-by-point responses associated with peer-review at Life Science Alliance will be published, alongside the manuscript. If you do want to opt out of having the reviewer reports and your point-by-point responses displayed, please let us know immediately.

IMPORTANT: If you will be unreachable at any time, please provide us with the email address of an alternate author. Failure to respond to routine queries may lead to unavoidable delays in publication.

Scheduling details will be available from our production department. You will receive proofs shortly before the publication date. Only essential corrections can be made at the proof stage so if there are any minor final changes you wish to make to the manuscript, please let the journal office know now.

DISTRIBUTION OF MATERIALS:
Authors are required to distribute freely any materials used in experiments published in Life Science Alliance. Authors are encouraged to deposit materials used in their studies to the appropriate repositories for distribution to researchers.

You can contact the journal office with any questions, contact@life-science-alliance.org

Again, congratulations on a very nice paper. I hope you found the review process to be constructive and are pleased with how the manuscript was handled editorially. We look forward to future exciting submissions from your lab.

Sincerely,

Sarita Hebbar, PhD
Scientific Editor
Life Science Alliance
http://www.lsajournal.org
